# Supplementary material for: Cell signaling model for arterial mechanobiology
Source: PLoS Comput Biol. 2020 Aug 24;16(8):e1008161. doi: 10.1371/journal.pcbi.1008161 (PMC7470387; doi:10.1371/journal.pcbi.1008161)
Supplement: S1 Fig — A subset of results from Fig 3, with a different scale for improved visualization of small changes. (PDF) [file pcbi.1008161.s001.pdf]

Supporting Information

Cell signaling model for arterial mechanobiology

Linda Irons, Jay D. Humphrey

Department of Biomedical Engineering, Yale University, New Haven, CT, USA

Corresponding author: linda.irons@yale.edu

S1 Fig. Sensitivity analysis for perturbed nodes

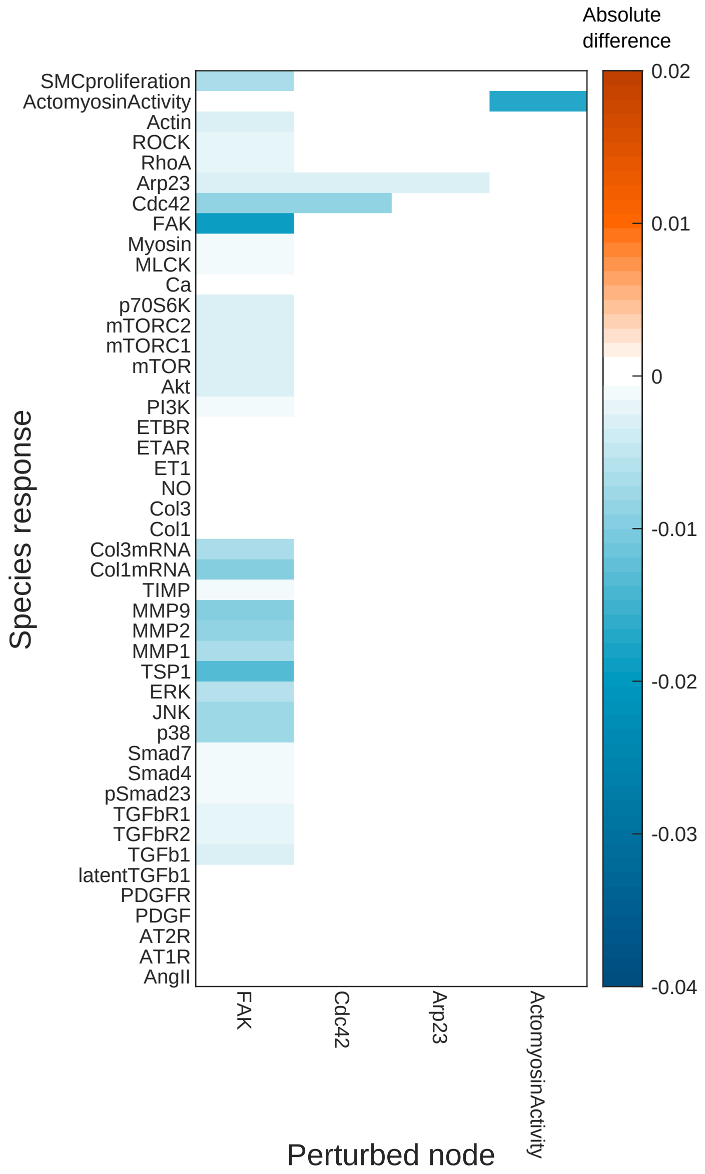

Figure : Subset of results from Fig 3 in the main text, showing the effect of attenuating FAK, Cdc42, Arp2/3 and ActomyosinActivity activity (via $Y_{max} = 0.1$, for each node in turn). The downstream effects of FAK are seen more clearly on this reduced scale, although the absolute differences remain small in magnitude due to low basal values (as discussed in the main text).
