# Supplementary material for: Cell signaling model for arterial mechanobiology
Source: PLoS Comput Biol. 2020 Aug 24;16(8):e1008161. doi: 10.1371/journal.pcbi.1008161 (PMC7470387; doi:10.1371/journal.pcbi.1008161)
Supplement: S2 Fig — TSP1 and TGFB1 responses to exogenous AngII under three levels of baseline stress, corresponding to Fig 4 in the main text. (PDF) [file pcbi.1008161.s002.pdf]

Supporting Information

Cell signaling model for arterial mechanobiology

Linda Irons, Jay D. Humphrey
Department of Biomedical Engineering, Yale University, New Haven, CT, USA
Corresponding author: linda.irons@yale.edu

S2 Fig. Additional species' responses to AngII

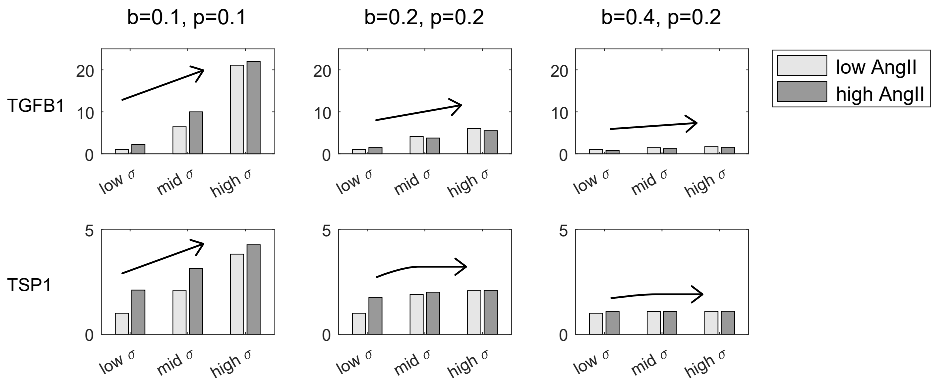

Figure : **Additional species' responses to exogenous AngII under three levels of baseline stress, corresponding to Fig 4 in the main text.** We show model outputs (relative to the baseline case, Stress=AngII=b) for four species of interest in response to three levels of stress, σ: low (b), intermediate ($b+p$), and high ($b+2p$), as well as low (b) and high ($b+2p$) AngII inputs. Arrows show general trends.
