# Supplementary material for: Cell signaling model for arterial mechanobiology
Source: PLoS Comput Biol. 2020 Aug 24;16(8):e1008161. doi: 10.1371/journal.pcbi.1008161 (PMC7470387; doi:10.1371/journal.pcbi.1008161)
Supplement: S3 Fig — An additional figure showing time-courses associated with the steady state model results in Fig 6. (PDF) [file pcbi.1008161.s003.pdf]

Supporting Information

Cell signaling model for arterial mechanobiology

Linda Irons, Jay D. Humphrey
Department of Biomedical Engineering, Yale University, New Haven, CT, USA
Corresponding author: linda.irons@yale.edu

S3 Fig. Time-courses of collagen mRNA levels

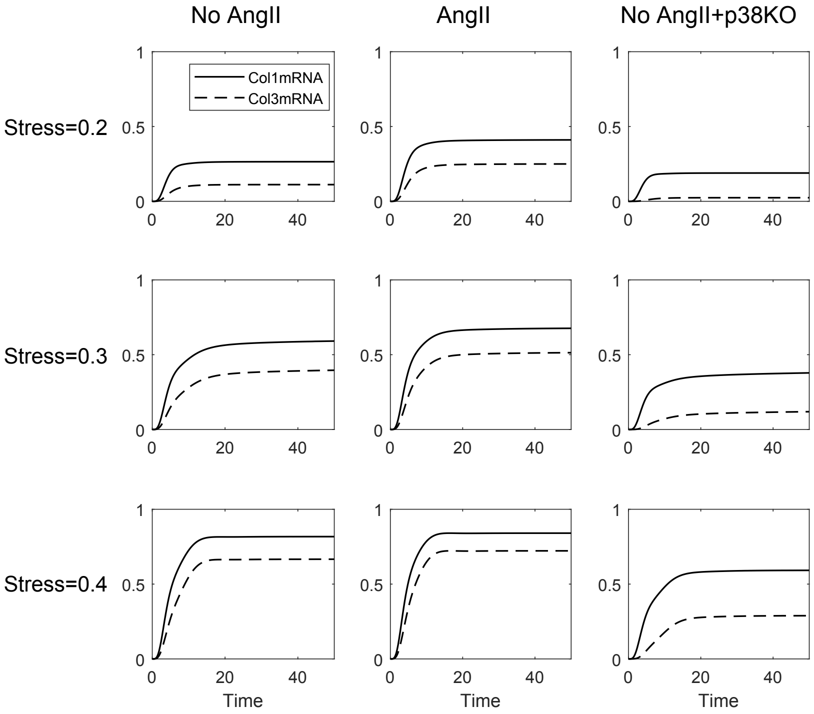

Figure : Simulated time-courses of collagen type I and collagen type III mRNA expression under three levels of stress ($y_{Stress} = \{0.2, 0.3, 0.4\}$) with and without exogenous AngII ($y_{AngIIin} = \{0, 0.2\}$), and without AngII but with a knockdown of p38MAPK (via a knockdown to 10% maximal activity). These correspond directly to the fold-change results presented in Fig 6 in the main text, where the baseline case is No AngII and Stress=0.2 (top left panel). Interestingly, although the fold change response of collagen type III mRNA to AngII was larger (Fig 6 in the main text), the absolute differences are similar. In the model, this finding is simply due to a lower basal value of collagen type III mRNA.
