# Supplementary material for: Cell signaling model for arterial mechanobiology
Source: PLoS Comput Biol. 2020 Aug 24;16(8):e1008161. doi: 10.1371/journal.pcbi.1008161 (PMC7470387; doi:10.1371/journal.pcbi.1008161)
Supplement: S4 Fig — An illustrative schematic of the bio-chemo-mechanical feedback system for tissue homeostasis. (PDF) [file pcbi.1008161.s004.pdf]

Supporting Information

Cell signaling model for arterial mechanobiology

Linda Irons, Jay D. Humphrey
Department of Biomedical Engineering, Yale University, New Haven, CT, USA
Corresponding author: linda.irons@yale.edu

S4 Fig. Vascular homeostasis

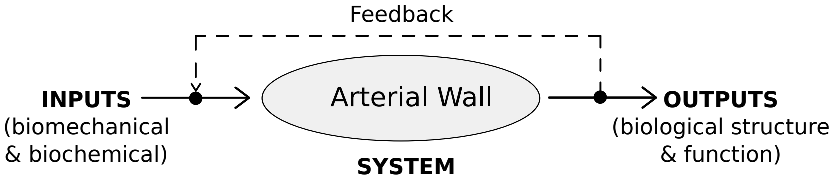

Figure : Basic bio-chemo-mechanical system for tissue homeostasis. Our network model determines the outputs generated by the model system in response to prescribed inputs. The indicated feedback represents changes due to G&R processes that would occur over longer timescales, which are not considered in the present model.
