# Supplementary material for: Cell signaling model for arterial mechanobiology
Source: PLoS Comput Biol. 2020 Aug 24;16(8):e1008161. doi: 10.1371/journal.pcbi.1008161 (PMC7470387; doi:10.1371/journal.pcbi.1008161)
Supplement: S1 Appendix — Species abbreviations, logic statements, and supporting references used in constructing the network structure shown in Fig 1. (PDF) [file pcbi.1008161.s005.pdf]

Supporting Information

Cell signaling model for arterial mechanobiology

Linda Irons, Jay D. Humphrey
Department of Biomedical Engineering, Yale University, New Haven, CT, USA
Corresponding author: linda.irons@yale.edu

S1 Appendix. Logic statements and supporting literature

List of 50 species and abbreviations used in the network diagram (Fig 1 in the main text) and in the logic-based rules (Table A).

Abbreviation	Description
Stress	Intramural Stress (pressure induced)
Wss	Wall shear stress (flow induced)
AngIIin	External Angiotensin II input
latentTGFB1	Latent TGFB1 complex
SACs	Stretch activated calcium channels
Integrins	Integrins
PDGF	Platelet derived growth factor
AngII	Angiotensin II
TGFB1	Transforming growth factor β-1
TGFBR2	TGFβ receptor II
TGFBR1	TGFβ receptor I
pSmad2/3	Phosphorylated Smad2/3 complex
Smad4	Smad4
Smad7	Smad7
TSP1	Thrombospondin-1
TIMP	Tissue inhibitor of metalloproteinase
p38	p38 MAPK (mitogen-activated protein kinase)
JNK	c-Jun N-terminal kinase
ERK	Extracellular signal–regulated kinase
MMP1	Matrix metalloproteinase-1
MMP2	Matrix metalloproteinase-2
MMP9	Matrix metalloproteinase-9
AT1R	Angiotensin receptor I
AT2R	Angiotensin receptor II
PDGFR	Platelet derived growth factor receptor
NO	Nitric Oxide
ET1	Endothelin-1
ETAR	Endothelin type A receptor
ETBR	Endothelin type B receptor
PI3K	Phosphoinositide 3-kinase
Akt	Akt
mTOR	Mechanistic target of rapamycin
mTORC1	mTOR complex 1 (mTOR, Raptor, . . .)
mTORC2	mTOR complex 2 (mTOR, Rictor, . . .)
p70S6K	p70S6K
Ca	Calcium
MLCK	Myosin light chain kinase

Myosin	Myosin
FAK	Focal adhesion kinase
Cdc42	Cdc42
Arp2/3	Arp2/3
RhoA	Rho-family GTPase
ROCK	Rho associated protein kinase
Actin	Actin
Col1mRNA	Collagen type I mRNA
Col3mRNA	Collagen type III mRNA
Col1	Collagen type I
Col3	Collagen type III
ActomyosinActivity	Actomyosin activity
SMCproliferation	Smooth muscle cell proliferation

We prescribe the dynamics of input nodes (Stress, Wss, AngIIin, SACs and Integrins) directly. Logic statements (Table A) govern the behavior of all other nodes. The 'AND' and 'NOT' operations are denoted by $\wedge$ and $\neg$ respectively, whereas 'OR' ($\vee$) relations are constructed afterwards by combining statements with common outputs. To illustrate the use of conditional operations, the statement "$A \wedge B \implies C$" represents a situation where both A and B are required to activate C. In contrast, "$A \vee B \implies C$" represents the case where either A or B can activate C independently, and "$A \wedge \neg B \implies C$" models activation of C by A, when inhibited by B. Note also the subtle difference between two ways of modeling inhibition using the 'NOT' ($\neg$) operation. Here, we use TGFBR2 $\wedge$ $\neg$Smad7 $\implies$ TGFBR1 instead of $\neg$Smad7 $\implies$ TGFBR1 to model inhibition of TGFBR1 by Smad7. The former describes Smad7 as suppressing an ongoing activation process, whereas the latter allows for spontaneous activation of TGFBR1 in the absence of Smad7. The most appropriate use of '$\neg$' should be considered on an individual basis.

Table A: Logic statements used to construct the normalized Hill ODEs. The 'AND' and 'NOT' operations are denoted by $\wedge$ and $\neg$ respectively, whereas 'OR' ($\vee$) relations are constructed afterwards by combining statements with common outputs.

Index	Rule	Experimental study
1	AngIIin $\implies$ AngII	Model input
2	Stress $\implies$ latentTGFB1	[1, 2]
3	latentTGFB1 $\wedge$ Stress $\wedge$ Integrins $\implies$ TGFB1	[3]
4	latentTGFB1 $\wedge$ MMP2 $\implies$ TGFB1	[4]
5	latentTGFB1 $\wedge$ MMP9 $\implies$ TGFB1	[5]
6	latentTGFB1 $\wedge$ TSP1 $\implies$ TGFB1	[6]
7	TGFB1 $\implies$ TGFBR2	[7, 8]
8	TGFBR2 $\wedge$ $\neg$Smad7 $\implies$ TGFBR1	[9, 10]
9	TGFBR1 $\implies$ pSmad2/3	[3, 11, 12]
10	pSmad2/3 $\implies$ Smad4	[13, 14]
11	Smad4 $\implies$ Smad7	[9]
12	Smad4 $\implies$ Col1mRNA	[15] (in Supplementary)
13	Smad4 $\implies$ Col3mRNA	[15] (in Supplementary)
14	Smad4 $\implies$ TIMP	[16]
15	TGFBR1 $\implies$ p38	[11]
16	PDGFR $\implies$ p38	[14]
17	p38 $\implies$ TSP1	[17]
18	p38 $\wedge$ $\neg$TIMP $\implies$ MMP1	[18]
19	p38 $\wedge$ $\neg$TIMP $\implies$ MMP2	[19]
20	p38 $\wedge$ $\neg$TIMP $\implies$ MMP9	[18, 20]

21	p38 $\implies$ Col1mRNA	[21, 22]
22	p38 $\implies$ Col3mRNA	[21]
23	TGFBR1 $\wedge \neg$AT2R $\implies$ ERK	[11, 13, 23]
24	PDGFR $\wedge \neg$AT2R $\implies$ ERK	[14, 23, 24]
25	ERK $\implies$ TSP1	[17, 25]
26	ERK $\wedge \neg$TIMP $\implies$ MMP2	[19, 26, 27]
27	ERK $\wedge \neg$TIMP $\implies$ MMP9	[20, 26]
28	ERK $\implies$ Col1mRNA	[28]
29	TGFBR1 $\implies$ JNK	[11]
30	PDGFR $\implies$ JNK	[14]
31	JNK $\implies$ TSP1	[17, 25]
32	JNK $\wedge \neg$TIMP $\implies$ MMP2	[29–31]
33	PDGF $\implies$ PDGFR	[32]
34	PDGFR $\implies$ PI3K	[24]
35	PI3K $\implies$ Akt	[24, 33]
36	Akt $\implies$ mTOR	[33]
37	mTOR $\implies$ mTORC1	[34]
38	mTOR $\implies$ mTORC2	[34]
39	mTORC2 $\implies$ Akt	[34]
40	mTORC2 $\wedge \neg$AT2R $\implies$ RhoA	[35–37]
41	mTORC1 $\implies$ p70S6K	[38]
42	p70S6K $\implies$ SMCproliferation	[38]
43	Col1mRNA $\wedge \neg$MMP1 $\wedge \neg$MMP2 $\implies$ Col1	[39]
44	Col3mRNA $\wedge \neg$MMP1 $\wedge \neg$MMP2 $\implies$ Col3	[39]
45	Wss $\implies$ NO	[40, 41]
46	Wss $\wedge \neg$NO $\implies$ ET1	[42]
47	ET1 $\implies$ ETAR	[43]
48	ETAR $\implies$ Ca	[44–46]
49	ETAR $\implies$ PI3K	[44]
50	ETAR $\wedge \neg$AT2R $\implies$ ERK	[47, 48]
51	ET1 $\implies$ ETBR	[43]
52	ETBR $\implies$ NO	[43, 49]
53	AngII $\implies$ AT1R	[23]
54	AngII $\implies$ AT2R	[23]
55	AT1R $\implies$ p38	[50]
56	AT1R $\wedge \neg$AT2R $\implies$ ERK	[23, 27, 51]
57	AT1R $\implies$ JNK	[52]
58	AT1R $\implies$ Ca	[53]
59	AT1R $\implies$ pSmad2/3	[51]
60	AT1R $\implies$ PI3K	[53]
61	AT2R $\implies$ NO	[54]
62	AT1R $\wedge \neg$AT2R $\implies$ RhoA	[52]
63	Ca $\wedge \neg$ NO $\implies$ MLCK	[55]
64	MLCK $\implies$ Myosin	[55]
65	Actin $\wedge$ Myosin $\implies$ ActomyosinActivity	[49, 55]
66	RhoA $\implies$ ROCK	[56]
67	ROCK $\implies$ Actin	[56]
68	ROCK $\wedge \neg$ NO $\implies$ MLCK	[56]
69	Stress $\wedge$ Integrins $\wedge \neg$AT2R $\implies$ RhoA	[57] (in Supplementary)
70	Stress $\wedge$ SACs $\implies$ Ca	[58]
71	Stress $\implies$ AngII	[1]
72	TGFBR1 $\implies$ PI3K	[59]

73	ERK $\implies$ SMCproliferation	[60]
74	Stress $\implies$ PDGF	[61, 62]
75	Akt $\wedge \neg$TIMP $\implies$ MMP2	[63]
76	Integrins $\wedge$ Stress $\implies$ FAK	[64–67]
77	FAK $\implies$ p38	[67, 68]
78	FAK $\wedge \neg$AT2R $\implies$ ERK	[64, 65, 67, 69]
79	FAK $\implies$ JNK	[70]
80	FAK $\implies$ Cdc42	[71]
81	Cdc42 $\implies$ Arp2/3	[72]
82	Arp2/3 $\implies$ Actin	[72]

Remark 1: Wall shear stress

Since we do not explicitly model endothelial cell signaling, the response to wall shear stress is simplified here. In accordance with experimental observations, endothelin-1 (ET1) should increase when the shear stress drops below its homeostatic level, and nitric oxide (NO) should increase when shear stress exceeds it [73]. Vasoconstriction and vasodilation then act, respectively, to modify the vessel radius and restore the homeostatic value of shear stress [74]. It is known that shear stress induces the activation of potassium channels, which upregulate eNOS and thereby NO [41, 75], but the direct effect of shear stress on ET1 is less clear. Several studies show an inhibitory effect of NO on ET1 [42, 76–78], however it is not yet clear if we should include a direct effect of shear stress on ET1, or if the inhibition by NO is sufficient to explain experimental observations. Three possibilities include a direct activation, direct suppression, or no direct effect. After testing each of these options, inclusion of activation provided the highest number of qualitative matches to the experimental input–output data (Fig 2A in the main text). This structure is also most consistent with a study in which gradual increases in shear stress were used, and a transient increase in ET1 was seen before its suppression [42]. An initial increase in ET1 is seen only when it is activated by shear stress but then inhibited by NO, not in the two alternative cases (no direct activation, or a direct suppression). In those cases, maximal concentrations of ET1 occurred in the static case ($\tau_w = 0$), and steadily decrease with increasing shear.

References

[1] Q. Li, Y. Muragaki, I. Hatamura, H. Ueno, and A. Ooshima. Stretch-induced collagen synthesis in cultured smooth muscle cells from rabbit aortic media and a possible involvement of angiotensin II and transforming growth factor-β. *Journal of Vascular Research*, 35(2):93–103, 1998.

[2] B. L. Riser, P. Cortes, C. Heilig, J. Grondin, S. Ladson-Wofford, D. Patterson, and R. G. Narins. Cyclic stretching force selectively up-regulates transforming growth factor-beta isoforms in cultured rat mesangial cells. *The American Journal of Pathology*, 148(6):1915, 1996.

[3] C. J. Turner, K. Badu-Nkansah, D. Crowley, A. van der Flier, and R. O. Hynes. $\alpha 5$ and αv integrins cooperate to regulate vascular smooth muscle and neural crest functions in vivo. *Development*, 142(4): 797–808, 2015.

[4] W. Xiong, T. Meisinger, R. Knispel, J. M. Worth, and B. T. Baxter. MMP-2 regulates Erk1/2 phosphorylation and aortic dilatation in Marfan syndrome. *Circulation Research*, 110(12):e92–e101, 2012.

[5] V. Lemaître, H. E. Kim, M. Forney-Prescott, Y. Okada, and J. D'Armiento. Transgenic expression of matrix metalloproteinase-9 modulates collagen deposition in a mouse model of atherosclerosis. *Atherosclerosis*, 205(1):107–112, 2009.

[6] C. W. Kim, A. Pokutta-Paskaleva, S. Kumar, L. H. Timmins, A. D. Morris, D.-W. Kang, S. Dalal, T. Chadid, K. M. Kuo, J. Raykin, et al. Disturbed flow promotes arterial stiffening through thrombospondin-1. *Circulation*, 136(13):1217–1232, 2017.

[7] Y. E. Zhang. Non-Smad pathways in TGF-β signaling. *Cell Research*, 19(1):128, 2009.

[8] A. M. Murphy, A. L. Wong, and M. Bezuhly. Modulation of angiotensin II signaling in the prevention of fibrosis. *Fibrogenesis & Tissue Repair*, 8(1):7, 2015.

[9] L. Zhu, S. Chen, and Y. Chen. Unraveling the biological functions of Smad7 with mouse models. *Cell & Bioscience*, 1(1):44, 2011.

[10] M. Bitzer, G. von Gersdorff, D. Liang, A. Dominguez-Rosales, A. A. Beg, M. Rojkind, and E. P. Böttinger. A mechanism of suppression of TGF–β/SMAD signaling by NF-κB/RelA. *Genes & Development*, 14(2): 187–197, 2000.

[11] U. Seay, D. Sedding, S. Krick, M. Hecker, W. Seeger, and O. Eickelberg. Transforming growth factor-β-dependent growth inhibition in primary vascular smooth muscle cells is p38-dependent. *Journal of Pharmacology and Experimental Therapeutics*, 315(3):1005–1012, 2005.

[12] W. Li, Q. Li, Y. Jiao, L. Qin, R. Ali, J. Zhou, J. Ferruzzi, R. W. Kim, A. Geirsson, H. C. Dietz, et al. Tgfbr2 disruption in postnatal smooth muscle impairs aortic wall homeostasis. *The Journal of Clinical Investigation*, 124(2):755–767, 2014.

[13] T. M. Holm, J. P. Habashi, J. J. Doyle, D. Bedja, Y. Chen, C. Van Erp, M. E. Lindsay, D. Kim, F. Schoenhoff, R. D. Cohn, et al. Noncanonical TGFβ signaling contributes to aortic aneurysm progression in Marfan syndrome mice. *Science*, 332(6027):358–361, 2011.

[14] X. Mao, P. DeBenedittis, Y. Sun, J. Chen, K. Yuan, K. Jiao, and Y. Chen. Vascular smooth muscle cell Smad4 gene is important for mouse vascular development. *Arteriosclerosis, Thrombosis, and Vascular Biology*, 32(9):2171–2177, 2012.

[15] P. Zhang, S. Hou, J. Chen, J. Zhang, F. Lin, R. Ju, X. Cheng, X. Ma, Y. Song, Y. Zhang, et al. Smad4 deficiency in smooth muscle cells initiates the formation of aortic aneurysm. *Circulation Research*, 118 (3):388–399, 2016.

[16] R. R. Díez, R. Rodrigues-Díez, C. Lavoz, S. Rayego-Mateos, E. Civantos, J. Rodríguez-Vita, S. Mezzano, A. Ortiz, J. Egido, and M. Ruiz-Ortega. Statins inhibit angiotensin II/Smad pathway and related vascular fibrosis, by a TGF-β-independent process. *PLoS One*, 5(11):e14145, 2010.

[17] F. C. McGillicuddy, D. O'Toole, J. A. Hickey, W. M. Gallagher, K. A. Dawson, and A. K. Keenan. TGF-β1-induced thrombospondin-1 expression through the p38 MAPK pathway is abolished by fluvastatin in human coronary artery smooth muscle cells. *Vascular Pharmacology*, 44(6):469–475, 2006.

[18] S. A. Potthoff, S. Stamer, K. Grave, E. Königshausen, S. H. Sivritas, M. Thieme, Y. Mori, M. Woznowski, L. C. Rump, and J. Stegbauer. Chronic p38 mitogen-activated protein kinase inhibition improves vascular function and remodeling in angiotensin II-dependent hypertension. *Journal of the Renin-Angiotensin-Aldosterone System*, 17(3):1470320316653284, 2016.

[19] Y. Cui, Y.-W. Sun, H.-S. Lin, W.-M. Su, Y. Fang, Y. Zhao, X.-Q. Wei, Y.-H. Qin, K. Kohama, and Y. Gao. Platelet-derived growth factor-BB induces matrix metalloproteinase-2 expression and rat vascular smooth muscle cell migration via ROCK and ERK/p38 MAPK pathways. *Molecular and Cellular Biochemistry*, 393(1-2):255–263, 2014.

[20] R. Forough, N. Koyama, D. Hasenstab, H. Lea, M. Clowes, S. T. Nikkari, and A. W. Clowes. Overexpression of tissue inhibitor of matrix metalloproteinase-1 inhibits vascular smooth muscle cell functions in vitro and in vivo. *Circulation Research*, 79(4):812–820, 1996.

[21] R. M. Touyz, G. He, M. El Mabrouk, and E. L. Schiffrin. p38 Map kinase regulates vascular smooth muscle cell collagen synthesis by angiotensin II in SHR but not in WKY. *Hypertension*, 37(2):574–580, 2001.

[22] M. Sato, D. Shegogue, E. A. Gore, E. A. Smith, M. Trojanowska, and P. J. McDermott. Role of p38 MAPK in transforming growth factor β stimulation of collagen production by scleroderma and healthy dermal fibroblasts. *Journal of Investigative Dermatology*, 118(4):704–711, 2002.

[23] J. P. Habashi, J. J. Doyle, T. M. Holm, H. Aziz, F. Schoenhoff, D. Bedja, Y. Chen, A. N. Modiri, D. P. Judge, and H. C. Dietz. Angiotensin II type 2 receptor signaling attenuates aortic aneurysm in mice through ERK antagonism. *Science*, 332(6027):361–365, 2011.

[24] N. Begum, S. Hockman, and V. C. Manganiello. Phosphodiesterase 3A (PDE3A) deletion suppresses proliferation of cultured murine vascular smooth muscle cells (VSMCs) via inhibition of mitogen-activated protein kinase (MAPK) signaling and alterations in critical cell cycle regulatory proteins. *Journal of Biological Chemistry*, 286(29):26238–26249, 2011.

[25] R. J. Chavez, R. M. Haney, R. H. Cuadra, R. Ganguly, R. K. Adapala, C. K. Thodeti, and P. Raman. Upregulation of thrombospondin-1 expression by leptin in vascular smooth muscle cells via JAK2-and MAPK-dependent pathways. *American Journal of Physiology-Cell Physiology*, 303(2):C179–C191, 2012.

[26] A. Ghosh, P. D. DiMusto, L. K. Ehrlichman, O. Sadiq, B. McEvoy, J. S. Futchko, P. K. Henke, J. L. Eliason, and G. R. Upchurch Jr. The role of extracellular signal-related kinase during abdominal aortic aneurysm formation. *Journal of the American College of Surgeons*, 215(5):668–680, 2012.

[27] Y. Zhang, J. C. Naggar, C. M. Welzig, D. Beasley, K. S. Moulton, H.-J. Park, and J. B. Galper. Simvastatin inhibits angiotensin II-induced abdominal aortic aneurysm formation in apolipoprotein E-knockout mice: possible role of ERK. *Arteriosclerosis, Thrombosis, and Vascular Biology*, 29(11):1764–1771, 2009.

[28] P.-L. Tharaux, C. Chatziantoniou, F. Fakhouri, and J.-C. Dussaule. Angiotensin II activates collagen I gene through a mechanism involving the MAP/ER kinase pathway. *Hypertension*, 36(3):330–336, 2000.

[29] W. P. Robinson III, C. D. Douillet, P. M. Milano, R. C. Boucher, C. Patterson, and P. B. Rich. ATP stimulates MMP-2 release from human aortic smooth muscle cells via JNK signaling pathway. *American Journal of Physiology-Heart and Circulatory Physiology*, 290(5):H1988–H1996, 2006.

[30] C. Wang, Q. Chang, X. Sun, X. Qian, P. Liu, H. Pei, X. Guo, and W. Liu. Angiotensin II induces an increase in matrix metalloproteinase 2 expression in aortic smooth muscle cells of ascending thoracic aortic aneurysms through JNK, ERK1/2, and p38 MAPK activation. *Journal of Cardiovascular Pharmacology*, 66(3):285–293, 2015.

[31] E. Jiménez, E. P. de la Blanca, L. Urso, I. González, J. Salas, and M. Montiel. Angiotensin II induces MMP 2 activity via FAK/JNK pathway in human endothelial cells. *Biochemical and Biophysical Research Communications*, 380(4):769–774, 2009.

[32] J. Schlessinger and A. Ullrich. Growth factor signaling by receptor tyrosine kinases. *Neuron*, 9(3):383–391, 1992.

[33] I. Shiojima and K. Walsh. Role of Akt signaling in vascular homeostasis and angiogenesis. *Circulation Research*, 90(12):1243–1250, 2002.

[34] Y. Jiao, G. Li, Q. Li, R. Ali, L. Qin, W. Li, Y. Qyang, D. M. Greif, A. Geirsson, J. D. Humphrey, et al. mTOR (mechanistic target of rapamycin) inhibition decreases mechanosignaling, collagen accumulation, and stiffening of the thoracic aorta in elastin-deficient mice. *Arteriosclerosis, Thrombosis, and Vascular Biology*, 37(9):1657–1666, 2017.

[35] L. Liu, Y. Luo, L. Chen, T. Shen, B. Xu, W. Chen, H. Zhou, X. Han, and S. Huang. Rapamycin inhibits cytoskeleton reorganization and cell motility by suppressing RhoA expression and activity. *Journal of Biological Chemistry*, 285(49):38362–38373, 2010.

[36] E. Jacinto, R. Loewith, A. Schmidt, S. Lin, M. A. Rüegg, A. Hall, and M. N. Hall. Mammalian tor complex 2 controls the actin cytoskeleton and is rapamycin insensitive. *Nature Cell Biology*, 6(11):1122, 2004.

[37] C. Savoia, T. Ebrahimian, Y. He, J.-P. Gratton, E. L. Schiffrin, and R. M. Touyz. Angiotensin II/AT2 receptor-induced vasodilation in stroke-prone spontaneously hypertensive rats involves nitric oxide and cGMP-dependent protein kinase. *Journal of Hypertension*, 24(12):2417–2422, 2006.

[38] W. Li, Q. Li, L. Qin, R. Ali, Y. Qyang, M. Tassabehji, B. R. Pober, W. C. Sessa, F. J. Giordano, and G. Tellides. Rapamycin inhibits smooth muscle cell proliferation and obstructive arteriopathy attributable to elastin deficiency. *Arteriosclerosis, Thrombosis, and Vascular Biology*, 33(5):1028–1035, 2013.

[39] H. Nagase, R. Visse, and G. Murphy. Structure and function of matrix metalloproteinases and TIMPs. *Cardiovascular Research*, 69(3):562–573, 2006.

[40] G. M. Buga, M. E. Gold, J. M. Fukuto, and L. J. Ignarro. Shear stress-induced release of nitric oxide from endothelial cells grown on beads. *Hypertension*, 17(2):187–193, 1991.

[41] M. Uematsu, Y. Ohara, J. P. Navas, K. Nishida, T. J. Murphy, R. W. Alexander, R. M. Nerem, and D. G. Harrison. Regulation of endothelial cell nitric oxide synthase mRNA expression by shear stress. *American Journal of Physiology-Cell Physiology*, 269(6):C1371–C1378, 1995.

[42] M.J. Kuchan and J.A. Frangos. Shear stress regulates endothelin-1 release via protein kinase C and cGMP in cultured endothelial cells. *American Journal of Physiology-Heart and Circulatory Physiology*, 264(1): H150–H156, 1993.

[43] N. Murakoshi, T. Miyauchi, Y. Kakinuma, T. Ohuchi, K. Goto, M. Yanagisawa, and I. Yamaguchi. Vascular endothelin-B receptor system in vivo plays a favorable inhibitory role in vascular remodeling after injury revealed by endothelin-B receptor–knockout mice. *Circulation*, 106(15):1991–1998, 2002.

[44] D. Chansel, M. Ciroldi, S. Vandermeersch, L. F. Jackson, A.-M. Gomez, D. Henrion, D. C. Lee, T. M. Coffman, S. Richard, J.-C. Dussaule, et al. Heparin binding EGF is necessary for vasospastic response to endothelin. *The FASEB Journal*, 20(11):1936–1938, 2006.

[45] M. K. Morris, J. Saez-Rodriguez, P. K. Sorger, and D. A. Lauffenburger. Logic-based models for the analysis of cell signaling networks. *Biochemistry*, 49(15):3216–3224, 2010.

[46] T. Enoki, S. Miwa, A. Sakamoto, T. Minowa, T. Komuro, S. Kobayashi, H. Ninomiya, and T. Masaki. Functional coupling of ETA receptor with Ca (2+)-permeable nonselective cation channel in mouse fibroblasts and rabbit aortic smooth-muscle cells. *Journal of Cardiovascular Pharmacology*, 26:S258–61, 1995.

[47] Q.-W. Chen, L. Edvinsson, and C.-B. Xu. Role of ERK/MAPK in endothelin receptor signaling in human aortic smooth muscle cells. *BMC Cell Biology*, 10(1):52, 2009.

[48] D. Chevalier, E. Thorin, and B. G. Allen. Simultaneous measurement of ERK, p38, and JNK MAP kinase cascades in vascular smooth muscle cells. *Journal of Pharmacological and Toxicological Methods*, 44(2): 429–439, 2000.

[49] M. P. Schneider, E. I. Boesen, and D. M. Pollock. Contrasting actions of endothelin ETA and ETB receptors in cardiovascular disease. *Annu. Rev. Pharmacol. Toxicol.*, 47:731–759, 2007.

[50] V. Subramanian, J. Golledge, E. B. Heywood, D. Bruemmer, and A. Daugherty. Regulation of PPARγ by Angiotensin II via TGF-β1 Activated p38 MAP Kinase in Aortic Smooth Muscle Cells. *Arteriosclerosis, Thrombosis, and Vascular Biology*, 32(2):397, 2012.

[51] S.-Q. Kuang, L. Geng, S. K. Prakash, J.-M. Cao, S. Guo, C. Villamizar, C. S. Kwartler, A. M. Peters, A. R. Brasier, and D. M. Milewicz. Aortic remodeling after transverse aortic constriction in mice is attenuated with AT1 receptor blockade. *Arteriosclerosis, Thrombosis, and Vascular Biology*, 33(9):2172–2179, 2013.

[52] S.-H. Tsai, P.-H. Huang, Y.-J. Peng, W.-C. Chang, H.-Y. Tsai, H.-B. Leu, J.-W. Chen, and S.-J. Lin. Zoledronate attenuates angiotensin II-induced abdominal aortic aneurysm through inactivation of Rho/ROCK-dependent JNK and NF-κB pathway. *Cardiovascular Research*, 100(3):501–510, 2013.

[53] C. Vecchione, E. Patrucco, G. Marino, L. Barberis, R. Poulet, A. Aretini, A. Maffei, M. T. Gentile, M. Storto, O. Azzolino, et al. Protection from angiotensin II–mediated vasculotoxic and hypertensive response in mice lacking PI3Kγ. *Journal of Experimental Medicine*, 201(8):1217–1228, 2005.

[54] H. M. Siragy, T. Inagami, T. Ichiki, and R. M. Carey. Sustained hypersensitivity to angiotensin II and its mechanism in mice lacking the subtype-2 (AT2) angiotensin receptor. *Proceedings of the National Academy of Sciences*, 96(11):6506–6510, 1999.

[55] F. Hong, B. D. Haldeman, D. Jackson, M. Carter, J. E. Baker, and C. R. Cremo. Biochemistry of smooth muscle myosin light chain kinase. *Archives of Biochemistry and Biophysics*, 510(2):135–146, 2011.

[56] M. Surma, L. Wei, and J. Shi. Rho kinase as a therapeutic target in cardiovascular disease. *Future Cardiology*, 7(5):657–671, 2011.

[57] D. Shen, J. Li, J. J. Lepore, T. J. T. Anderson, S. Sinha, A. Y. Lin, L. Cheng, E. D. Cohen, J. D. Roberts Jr, S. Dedhar, et al. Aortic aneurysm generation in mice with targeted deletion of integrin-linked kinase in vascular smooth muscle cells. *Circulation research*, 109(6):616–628, 2011.

[58] K. Muraki, Y. Iwata, Y. Katanosaka, T. Ito, S. Ohya, M. Shigekawa, and Y. Imaizumi. TRPV2 is a component of osmotically sensitive cation channels in murine aortic myocytes. *Circulation Research*, 93 (9):829–838, 2003.

[59] S. Lamouille and R. Derynck. Cell size and invasion in TGF-β–induced epithelial to mesenchymal transition is regulated by activation of the mTOR pathway. *The Journal of Cell Biology*, 178(3):437–451, 2007.

[60] P. A Suwanabol, S. M Seedial, X. Shi, F. Zhang, D. Yamanouchi, D. Roenneburg, B. Liu, and K. C. Kent. Transforming growth factor-β increases vascular smooth muscle cell proliferation through the Smad3 and extracellular signal-regulated kinase mitogen-activated protein kinases pathways. *Journal of Vascular Surgery*, 56(2):446–454, 2012.

[61] E. Wilson, Q. Mai, K. Sudhir, R. H. Weiss, and H. E. Ives. Mechanical strain induces growth of vascular smooth muscle cells via autocrine action of PDGF. *The Journal of Cell Biology*, 123(3):741–747, 1993.

[62] Y. Hu, G. Böck, G. Wick, and Q. Xu. Activation of PDGF receptor α in vascular smooth muscle cells by mechanical stress. *The FASEB Journal*, 12(12):1135–1142, 1998.

[63] K. W. Seo, S. J. Lee, Y. H. Kim, J. U. Bae, S. Y. Park, S. S. Bae, and C. D. Kim. Mechanical stretch increases MMP-2 production in vascular smooth muscle cells via activation of PDGFR-β/Akt signaling pathway. *PLoS One*, 8(8):e70437, 2013.

[64] S. Albinsson and P. Hellstrand. Integration of signal pathways for stretch-dependent growth and differentiation in vascular smooth muscle. *American Journal of Physiology-Cell Physiology*, 293(2):C772–C782, 2007.

[65] S. Lehoux, B. Esposito, R. Merval, and A. Tedgui. Differential regulation of vascular focal adhesion kinase by steady stretch and pulsatility. *Circulation*, 111(5):643–649, 2005.

[66] O. Yamashita, K. Yoshimura, A. Nagasawa, K. Ueda, N. Morikage, Y. Ikeda, and K. Hamano. Periostin links mechanical strain to inflammation in abdominal aortic aneurysm. *PLoS One*, 8(11):e79753, 2013.

[67] J. G. Wang, M. Miyazu, E. Matsushita, M. Sokabe, and K. Naruse. Uniaxial cyclic stretch induces focal adhesion kinase (FAK) tyrosine phosphorylation followed by mitogen-activated protein kinase (MAPK) activation. *Biochemical and Biophysical Research Communications*, 288(2):356–361, 2001.

[68] R. Aikawa, T. Nagai, S. Kudoh, Y. Zou, M. Tanaka, M. Tamura, H. Akazawa, H. Takano, R. Nagai, and I. Komuro. Integrins play a critical role in mechanical stress–induced p38 MAPK activation. *Hypertension*, 39(2):233–238, 2002.

[69] G. Govindarajan, D. M. Eble, P. A. Lucchesi, and A. M. Samarel. Focal adhesion kinase is involved in angiotensin II–mediated protein synthesis in cultured vascular smooth muscle cells. *Circulation Research*, 87(8):710–716, 2000.

[70] L. J. Sundberg, L. M. Galante, H. M. Bill, C. P. Mack, and J. M. Taylor. An endogenous inhibitor of focal adhesion kinase blocks Rac1/JNK but not Ras/ERK-dependent signaling in vascular smooth muscle cells. *Journal of Biological Chemistry*, 278(32):29783–29791, 2003.

[71] C. E. Turner. Paxillin and focal adhesion signalling. *Nature Cell Biology*, 2(12):E231, 2000.

[72] L. E. Ma, R. Rohatgi, and M. W. Kirschner. The Arp2/3 complex mediates actin polymerization induced by the small GTP-binding protein Cdc42. *Proceedings of the National Academy of Sciences*, 95(26): 15362–15367, 1998.

[73] J. D. Humphrey. Vascular adaptation and mechanical homeostasis at tissue, cellular, and sub-cellular levels. *Cell Biochemistry and Biophysics*, 50(2):53–78, 2008.

[74] S. Rodbard. Vascular caliber. *Cardiology*, 60(1):4–49, 1975.

[75] S.-P. Olesen, D. Clapham, and P. Davies. Haemodynamic shear stress activates a K+ current in vascular endothelial cells. *Nature*, 331(6152):168, 1988.

[76] S. Kourembanas, L. P. McQuillan, G. K. Leung, and D. V. Faller. Nitric oxide regulates the expression of vasoconstrictors and growth factors by vascular endothelium under both normoxia and hypoxia. *The Journal of Clinical Investigation*, 92(1):99–104, 1993.

[77] H. Morawietz, R. Talanow, M. Szibor, U. Rueckschloss, A. Schubert, B. Bartling, D. Darmer, and J. Holtz. Regulation of the endothelin system by shear stress in human endothelial cells. *The Journal of Physiology*, 525(3):761–770, 2000.

[78] L. K. Kelly, S. Wedgwood, R. H. Steinhorn, and S. M. Black. Nitric oxide decreases endothelin-1 secretion through the activation of soluble guanylate cyclase. *American Journal of Physiology-Lung Cellular and Molecular Physiology*, 286(5):L984–L991, 2004.
