# Supplementary material for: Cell signaling model for arterial mechanobiology
Source: PLoS Comput Biol. 2020 Aug 24;16(8):e1008161. doi: 10.1371/journal.pcbi.1008161 (PMC7470387; doi:10.1371/journal.pcbi.1008161)
Supplement: S2 Appendix — Description and supporting figures for the process of selecting the optimal default Hill parameters. (PDF) [file pcbi.1008161.s006.pdf]

Supporting Information

Cell signaling model for arterial mechanobiology

Linda Irons, Jay D. Humphrey
Department of Biomedical Engineering, Yale University, New Haven, CT, USA
Corresponding author: linda.irons@yale.edu

S2 Appendix. Selection of default parameters

We first assume that the reaction weights and Hill parameters (w_i, n, EC_{50}) are uniform across the network [1–5] (*i.e.* identical values for each reaction). Additionally, let $w_i = 1$ for each reaction. The strength and functional forms for activation and inhibition are therefore identical for each reaction, as in Boolean models. This assumption captured qualitative network behaviors reasonably well in previous studies [1–5].

We conducted a parameter sweep for the Hill parameters (n and EC_{50}) and quantified the percentage of input–output relations that qualitatively matched between model and experiment (Fig A). Conflicting experimental results (Fig 2A in the main text) were not included at this stage, but were considered individually later. In addition to the Hill parameters, key factors influencing the qualitative results are the (uniform) baseline and perturbed values of the inputs, denoted by b and $b + p$, respectively, where p is the magnitude of the perturbation (Eqs 1–4 in the main text). We therefore consider the percentages of qualitative agreements between model and experiment for different combinations of four parameters: n, EC_{50}, b and p (Fig A), where a change in activity is deemed significant if there is an absolute difference of 0.05 (5% of maximal activity) or above relative to the corresponding steady state value at baseline.

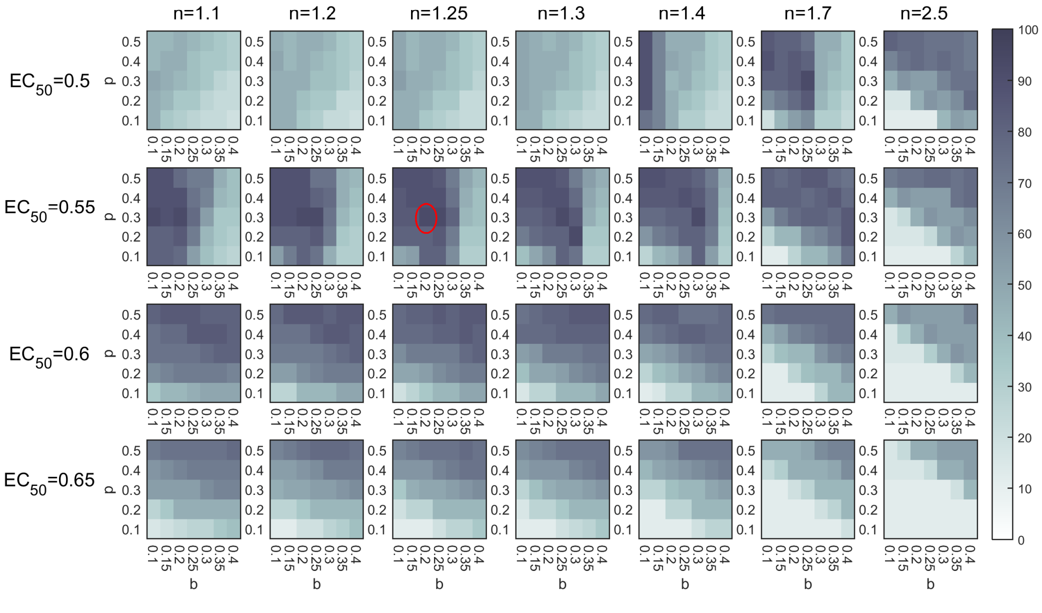

Figure A: Percentage (greyscale) of qualitative matches between the model and the (non-conflicting) experimental observations in Fig 2A in the main text. This parameter sweep was used to optimize the model with respect to four parameters: the basal input level, $b \in [0, 1]$, the magnitude of input perturbations, $p \in [0, 1]$, and the Hill parameters, $n > 0$ and $EC_{50} \in [0, 1]$ (Eq 5 in the main text). A change in activity is recorded if there is an absolute difference of 0.05 (5% of maximal activity) or above from baseline. We selected $n = 1.25$, $EC_{50} = 0.55$, $b = 0.2$ and $p = 0.3$ (red circle) as default parameters.

A general observation is that high n and high EC_{50} lead to weak signal propagation through the network; steeper sigmoids (higher n) require a higher threshold for activation while $EC_{50} > 0.5$

dampens signals along linear cascades (see S5 Appendix). Higher baseline and perturbation values can compensate for this, but in general weak signal propagation results in poorer matches to the experimental observations. Conversely, low n and low EC_{50} lead to strong signal propagation through the network, which saturates activity levels, making further perturbations ineffective. Despite some compensation between the values of n and EC_{50} (increasing EC_{50} is reversed somewhat by a decreased n and vice versa), based on Fig A, we selected $n = 1.25$, $EC_{50} = 0.55$, $b = 0.2$ and $p = 0.3$ as default parameters.

Next, consider the consistency of qualitative responses under changes in b and p. Fig B shows the percentage of increases or decreases for each of the 35 (b, p) pairs in Fig A with $n = 1.25$ and $EC_{50} = 0.55$. For many of the pairs, there is either always or never an increase in output; however, intermediate values highlight differential responses that are sensitive to input or perturbation levels. Examples include changes in MMPs, actomyosin activity, and SMC proliferation in response to increased stress or exogenous AngII, and changes in TGFβ1 in response to exogenous AngII. For these examples, the alternative response is decreased activity (Fig B). For others, such as Akt and p70S6K responses to Stress, no decreases are seen and the alternative is therefore that there is no significant change (where an absolute change must be over 0.05 to be deemed significant).

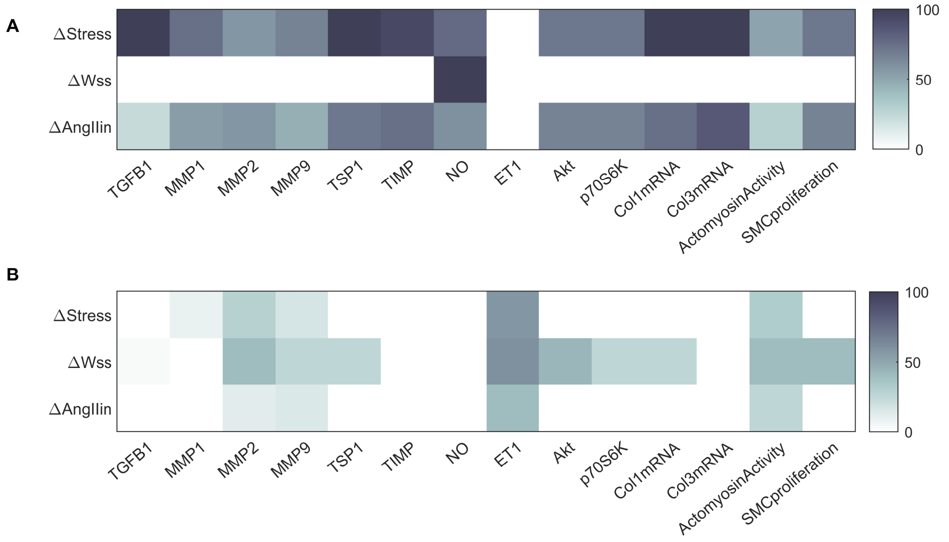

Figure B: Percentages (greyscale) of (A) increases and (B) decreases (relative to baseline) for each of the 35 (b, p) pairs (Fig A) for uniform Hill parameters $n = 1.25$ and $EC_{50} = 0.55$. Values other than 0 or 100 indicate sensitivity to input or perturbations levels; differential responses are present if there are both increases and decreases. The other possible outcome is no detectable change (using a threshold of 0.05).

References

[1] M. J. Kraeutler, A. R. Soltis, and J. J. Saucerman. Modeling cardiac β-adrenergic signaling with normalized-Hill differential equations: comparison with a biochemical model. *BMC Systems Biology*, 4 (1):157, 2010.

[2] K. A. Ryall, D. O. Holland, K. A. Delaney, M. J. Kraeutler, A. J. Parker, and J. J. Saucerman. Network reconstruction and systems analysis of cardiac myocyte hypertrophy signaling. *Journal of Biological Chemistry*, 287(50):42259–42268, 2012.

[3] P. M. Tan, K. S. Buchholz, J. H. Omens, A. D. McCulloch, and J. J. Saucerman. Predictive model identifies key network regulators of cardiomyocyte mechano-signaling. *PLoS Computational Biology*, 13 (11):e1005854, 2017.

[4] A. C. Zeigler, W. J. Richardson, J. W. Holmes, and J. J. Saucerman. A computational model of cardiac fibroblast signaling predicts context-dependent drivers of myofibroblast differentiation. *Journal of Molecular and Cellular Cardiology*, 94:72–81, 2016.

[5] J. Cursons, J. Gao, D. G. Hurley, P. R. Dunbar, M. D. Jacobs, E. J. Crampin, et al. Regulation of ERK-MAPK signaling in human epidermis. *BMC Systems Biology*, 9(1):41, 2015.
