# Supplementary material for: Cell signaling model for arterial mechanobiology
Source: PLoS Comput Biol. 2020 Aug 24;16(8):e1008161. doi: 10.1371/journal.pcbi.1008161 (PMC7470387; doi:10.1371/journal.pcbi.1008161)
Supplement: S3 Appendix — Fold-change responses and absolute activity of several species of interest as baseline and perturbation magnitudes vary. We show that non-monotonic responses to inputs underlie conflicting fold-change responses. (PDF) [file pcbi.1008161.s007.pdf]

Supporting Information

Cell signaling model for arterial mechanobiology

Linda Irons, Jay D. Humphrey
Department of Biomedical Engineering, Yale University, New Haven, CT, USA
Corresponding author: linda.irons@yale.edu

S3 Appendix. Sensitivity of fold-change responses to baseline conditions

We consider the consistency of qualitative responses under changes in b and p (S2 Appendix), highlighting differential responses that are sensitive to inputs or levels of perturbation. Examples include predicted changes in MMPs, actomyosin activity, and SMC proliferation in response to prescribed increases in stress or exogenous AngII, and predicted changes in TGFβ1 in response to prescribed exogenous AngII. For a subset of these cases, we illustrate this dependence by plotting fold changes in steady state behavior relative to the baseline ($p = 0$) case, as the parameters b and p vary, where p is a stress perturbation (shown below). Compared to no change (a fold change of unity, black grid), this illustrates cases where increases (TGFβ1, TSP1, NO) and decreases (ET1) are found consistently or where both increases and decreases (MMP1, MMP2) can be seen.

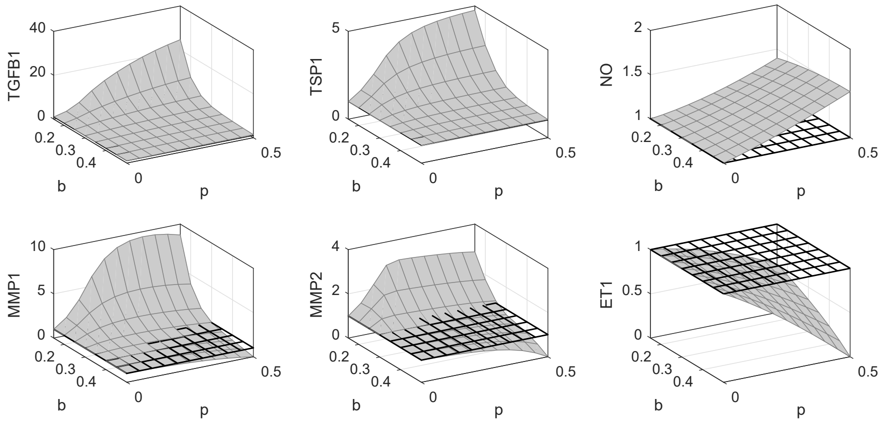

Figure A: **Sensitivity of fold-change measurements to baseline values and magnitudes of perturbation.** We show fold-changes relative to baseline of 6 (of the 50) species of interest as a function of basal input levels, $b \in [0.2, 0.5]$, and stress perturbation magnitude, $p \in [0, 0.5]$, for $n = 1.25$ and $EC_{50} = 0.55$. The black grid denotes no change (a fold-change of unity), and cases where either relative increases and decreases emerged (MMP1, MMP2) demonstrate a sensitivity of the qualitative outcome to baseline conditions and perturbation magnitude.

The cases for which there are differences in qualitative response can be understood better when plotting the behavior as a function of b and p in absolute terms (shown below), where we found that the species exhibiting inconsistent qualitative responses exhibited non-monotonic behavior as b and p increase. Qualitative conclusions thus depend on input level, that is, the baseline or point of reference for the comparison, and the magnitude of the perturbation. This is also illustrated clearly in a simple example (S4 Appendix) in which a non-monotonic input–output relation between TGFβ1 and MMPs occurred due to TIMP inhibition.

These results highlight the importance of carefully controlling or measuring baseline conditions. In order to characterize more complex (possibly non-monotonic) behaviors, which can arise in systems with feedback and inhibition, multiple perturbation magnitudes or pairings of baselines and perturbations should also be considered, rather than a single perturbation, which is often the case when

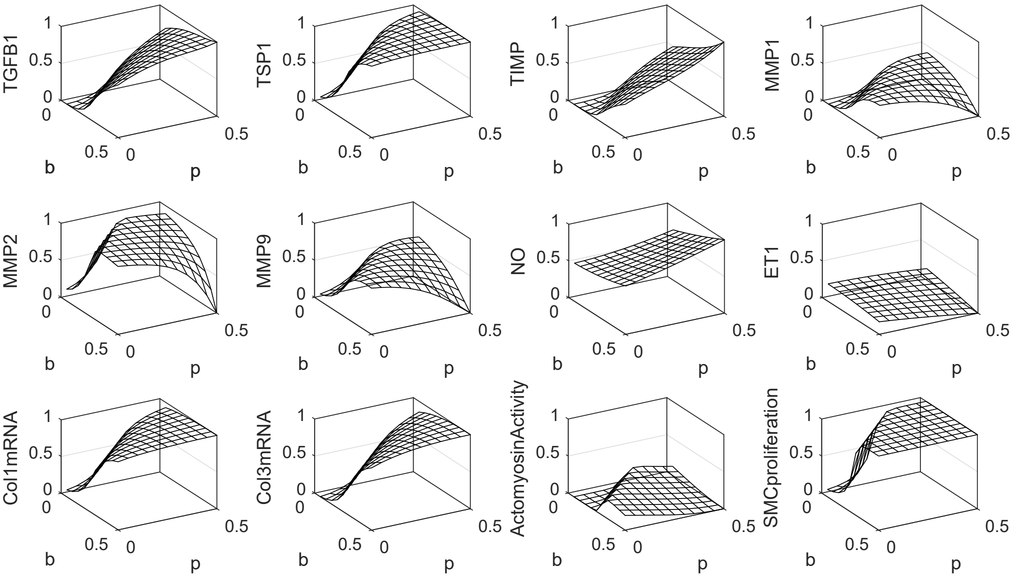

Figure B: **Absolute changes in steady state behavior of 12 species of interest as a function of basal input levels, $b \in [0, 0.5]$, and stress perturbation magnitude, $p \in [0, 0.5]$.** These correspond to the fold changes shown above, as well as for some additional species. The species that exhibit inconsistent fold-change responses are seen to have non-monotonic behavior as b and p increase, leading to a sensitivity to the baseline, or point of reference, for the comparison, as well as sensitivity to the magnitude of perturbation. Default parameters are $n = 1.25$ and $EC_{50} = 0.55$. Species values tending to 1 imply maximal activity; values tending to 0 imply no activity.

fold-changes are reported. Non-monotonic input–output relations could explain conflicting experimental findings, which often only consider single doses and baselines, therefore only sampling one point from this surface of possible fold-changes. Ruddy *et al.* [1] address this need in murine thoracic aortic rings under three states of tension, with and without exogenous AngII. The authors showed that, under low tension, MT1-MMP and MMP9 promoter expression were increased by AngII, yet with a high baseline tension there were significant decreases in promoter activity with the addition of AngII. This case study is discussed further in the main text, where we simulate AngII perturbations under different baseline levels of stress.

References

[1] J. M. Ruddy, J. A. Jones, R. E. Stroud, R. Mukherjee, F. G. Spinale, and J. S. Ikonomidis. Differential effects of mechanical and biological stimuli on matrix metalloproteinase promoter activation in the thoracic aorta. *Circulation*, 120(11 suppl 1):S262–S268, 2009.
