# Supplementary material for: Cell signaling model for arterial mechanobiology
Source: PLoS Comput Biol. 2020 Aug 24;16(8):e1008161. doi: 10.1371/journal.pcbi.1008161 (PMC7470387; doi:10.1371/journal.pcbi.1008161)
Supplement: S4 Appendix — An illustrative model used to demonstrate the process of formulating logic statements, generating the corresponding system of normalized Hill ODEs, and calculating the system steady states. In this example, we show that inhibition can lead to a non-monotonic input–output relation, and we illustrate how conflicting fold-change measurements can result. (PDF) [file pcbi.1008161.s008.pdf]

Supporting Information

Cell signaling model for arterial mechanobiology

Linda Irons, Jay D. Humphrey
Department of Biomedical Engineering, Yale University, New Haven, CT, USA
Corresponding author: linda.irons@yale.edu

S4 Appendix. Simple illustrative model

Consider a simple system, with activation and inhibition, in the schematic drawing below. We show the illustrative network in diagrammatic and rule-based form.

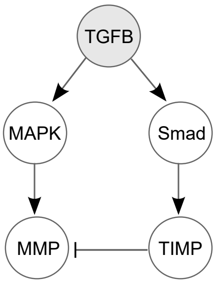

Inputs: TGFB, with basal value: b∈[0,1]

Logic statements:

TGFB ⇒ MAPK
TGFB ⇒ Smad
MAPK∧¬TIMP ⇒ MMP
Smad ⇒ TIMP

Governing equations for the example above take the form

$$y_1 = b, \tag{A1}$$

$$\frac{dy_2}{dt} = \frac{1}{\tau_2}\left(w_1 \frac{\beta y_1^n}{K^n + y_1^n} Y_{2_{max}} - y_2\right), \tag{A2}$$

$$\frac{dy_3}{dt} = \frac{1}{\tau_3}\left(w_2 \frac{\beta y_1^n}{K^n + y_1^n} Y_{3_{max}} - y_3\right), \tag{A3}$$

$$\frac{dy_4}{dt} = \frac{1}{\tau_4}\left(w_3 \left(1 - \frac{\beta y_5^n}{K^n + y_5^n}\right) \frac{\beta y_2^n}{K^n + y_2^n} Y_{4_{max}} - y_4\right), \tag{A4}$$

$$\frac{dy_5}{dt} = \frac{1}{\tau_5}\left(w_4 \frac{\beta y_3^n}{K^n + y_3^n} Y_{5_{max}} - y_5\right), \tag{A5}$$

where y_1–y_5 denote TGFB, MAPK, Smad, MMP, and TIMP, respectively, and $b \in [0, 1]$ is a scalar input level. The reaction weight for reaction i is denoted w_i, and the constant τ_j is a timescale associated with the decay of variable j. An explicit first order decay term represents more accurately the finite lifetimes of biological activity compared to Boolean models, in which decay occurs as a switch as soon as the activating species is removed. Here the decay parameter also scales the production term; this is derived in a few simple steps in [1], but arises due to normalization of each species to $[0, 1]$. Finally, we use the parameters $Y_{j_{max}} \in [0, 1]$ to allow external interventions such as full or partial knockdowns of a species; these constants control the fraction of the maximum activity level that can be attained.

Consider a simplifying assumption, in which $w_i = 1$, $\tau_j = 1$ and $Y_{j_{max}} = 1$. The equations become

$$y_1 = b, \tag{A6}$$

$$\frac{dy_2}{dt} = \frac{\beta y_1^n}{K^n + y_1^n} - y_2, \tag{A7}$$

$$\frac{dy_3}{dt} = \frac{\beta y_1^n}{K^n + y_1^n} - y_3, \tag{A8}$$

$$\frac{dy_4}{dt} = \left(1 - \frac{\beta y_5^n}{K^n + y_5^n}\right) \frac{\beta y_2^n}{K^n + y_2^n} - y_4, \tag{A9}$$

$$\frac{dy_5}{dt} = \frac{\beta y_3^n}{K^n + y_3^n} - y_5, \tag{A10}$$

for which the steady states are given by

$$\tilde{y}_1 = b, \tag{A11}$$

$$\tilde{y}_2 = \tilde{y}_3 = \frac{\beta b^n}{K^n + b^n} \tag{A12}$$

$$\tilde{y}_4 = \left(1 - \frac{\beta \alpha^n}{K^n + \alpha^n}\right) \alpha, \tag{A13}$$

$$\tilde{y}_5 = \alpha, \tag{A14}$$

where $\alpha = \beta \tilde{y}_2^n/(K^n + \tilde{y}_2^n) = \beta \tilde{y}_3^n/(K^n + \tilde{y}_3^n) \in [0, 1]$. Steady state solutions as a function of b are shown in Figure A, where we observe monotonocity in each species except MMPs, which first increase and then decrease for large inputs and correspondingly high levels of TIMP. The observation that inhibition can lead to a non-monotonic response to an input is interesting when it comes to interpreting experimental data, which are often presented as a fold change in activity (as, for example, determined from Western blotting). Typically, activity levels are compared for two values of the input (a baseline value and a perturbed value), and the conclusion is stated qualitatively as an 'increase' or 'decrease' in response to the stimulus. For outputs that respond monotonically to an input, this measure will be consistent when comparing activity across different baseline and perturbed levels of the input. In contrast, if the input–output relationship is non-monotonic (as for the MMPs in Fig A), opposing qualitative observations could occur for different baselines and perturbations. Consider, for illustrative purposes, the difference in output between points (i) and (ii) compared to (i) and (iii) in Fig B. This result highlights the need for careful control of baseline conditions in experiments and, importantly, suggests that multiple doses of exogenous inputs are necessary to fully characterize input–output relationships. This is likely to be important when dealing with larger signaling networks, where increasingly complex (and not necessarily monotonic) responses are expected.

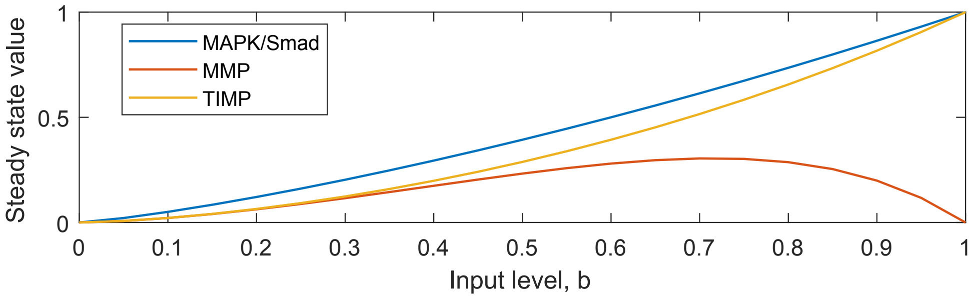

Figure A: Steady state output values of MAPK, Smad, MMP and TIMP (Eqs A12–A14) as a function of the input TGFB value, b. Hill parameters used were $n = 1.25$, $EC_{50} = 0.6$ and $w = 1$.

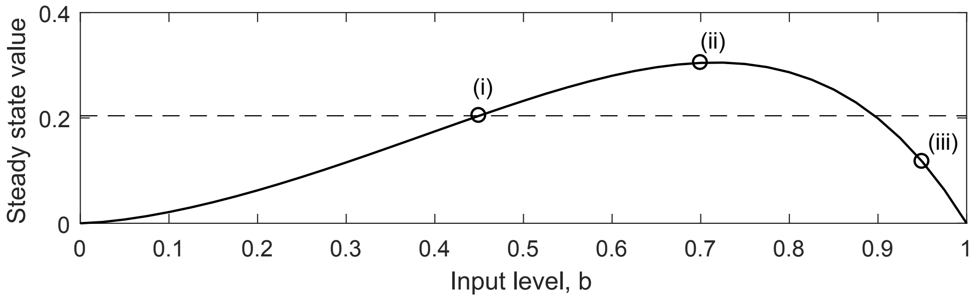

Figure B: Illustrative example to demonstrate that a non-monotonic input–output relationship (such as that for MMPs) can lead to conflicting qualitative results when sampling only two points (a baseline and single perturbed value). Measuring the output at (i) and (ii) would suggest that activity increases with input, whereas the opposite would be concluded for (i) and (iii). Two samples are therefore not sufficient to fully characterize the behavior.

References

[1] D. M. Wittmann, J. Krumsiek, J. Saez-Rodriguez, D. A. Lauffenburger, S. Klamt, and F. J. Theis. Transforming Boolean models to continuous models: methodology and application to T-cell receptor signaling. *BMC Systems Biology*, 3(1):98, 2009.
