# Supplementary material for: Cell signaling model for arterial mechanobiology
Source: PLoS Comput Biol. 2020 Aug 24;16(8):e1008161. doi: 10.1371/journal.pcbi.1008161 (PMC7470387; doi:10.1371/journal.pcbi.1008161)
Supplement: S5 Appendix — We demonstrate the role of Hill parameters in signal propagation, focusing on a linear cascade. We show how the choice of EC50 can either lead to decay, amplification, or preservation of signal strength. (PDF) [file pcbi.1008161.s009.pdf]

Supporting Information

Cell signaling model for arterial mechanobiology

Linda Irons, Jay D. Humphrey
Department of Biomedical Engineering, Yale University, New Haven, CT, USA
Corresponding author: linda.irons@yale.edu

S5 Appendix. Sensitivity to Hill parameters

Example: linear cascade
We consider a simple linear signaling cascade below where an input signal is represented by node A.

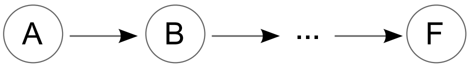

Linear signaling cascade, with input A.

Setting the weights and $Y_{max} = 1$, the governing equation for each species $Y \in \{B, ..., F\}$ is simply

$$\frac{dY}{dt} = \frac{1}{\tau_Y}\left(F(X) - Y\right), \tag{A1}$$

where X is the activating species, $F(X)$ is the Hill activation function described in Eq 5 (in the main text) and τ_Y is a timescale associated with decay of the variable Y.

We show in Fig A how the activity levels of nodes A–F are affected by three different values of EC_{50}, selected to represent the cases (i) $EC_{50} < 0.5$, (ii) $EC_{50} = 0.5$ and (iii) $EC_{50} > 0.5$. In these three cases, different qualitative outcomes emerge when there is a sustained input of $A = 0.5$: amplification, conservation, or attenuation of the signal. The value of EC_{50} thus controls more than just the half maximal activation; it also determines the extent to which further signal propagation can be sustained.

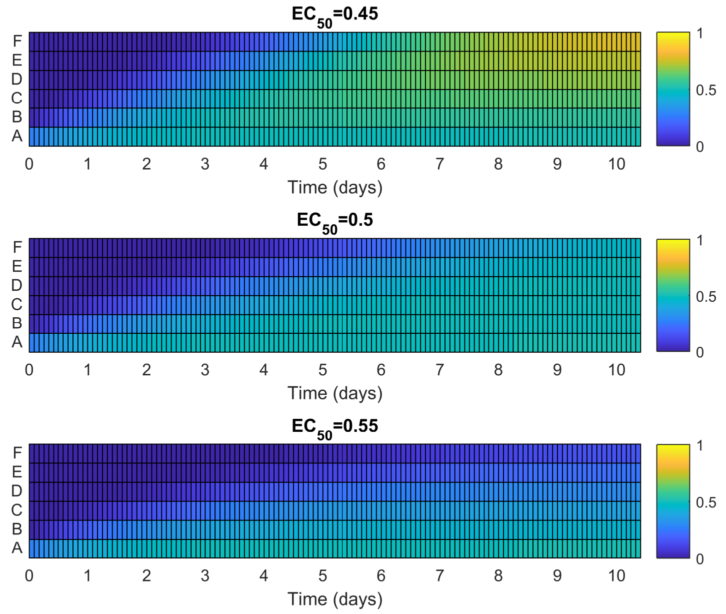

Figure A: Activity levels in a linear cascade, with input A reaching a value of 0.5. Here, the value of EC_{50} determines whether the signal is amplified ($EC_{50} < 0.5$), conserved ($EC_{50} = 0.5$) or attenuated ($EC_{50} > 0.5$).

The behaviors above can be understood from Eq A1. The steady state of this equation, where we use A and B instead of X and Y, is given by $B = F(A) = F(0.5)$. Due to monotonicity, we have that $F(0.5) > 0.5$ for $EC_{50} < 0.5$, meaning that the steady value of B is $B > 0.5$. As the Hill function is concave after $F(EC_{50})$, we find that $C = F(B) > B, \ldots$ and similarly for all further terms in the chain, resulting in signal amplification. Cases for conservation and attenuation (with $EC_{50} = 0.5$ and $EC_{50} > 0.5$, respectively) are similar.

In initial simulations, $EC_{50} = 0.5$ is a sensible starting point, but tuning this parameter can be useful when fitting experimental data. As an example, values of $EC_{50} = 0.5$ ([1–4]) and $EC_{50} = 0.6$ ([5]) have been used, with the larger value reflecting a tendency, overall, of a linear signal cascade to be attenuated. This effect is also influenced by the choice of Hill exponent, n, being less pronounced for Hill functions that are approximately linear (i.e. small n). As n increases, however, the function becomes closer to a step function (as in a Boolean description), and values quickly decay or amplify toward 0 or 1.

References

[1] M. J. Kraeutler, A. R. Soltis, and J. J. Saucerman. Modeling cardiac β-adrenergic signaling with normalized-Hill differential equations: comparison with a biochemical model. *BMC Systems Biology*, 4 (1):157, 2010.

[2] K. A. Ryall, D. O. Holland, K. A. Delaney, M. J. Kraeutler, A. J. Parker, and J. J. Saucerman. Network reconstruction and systems analysis of cardiac myocyte hypertrophy signaling. *Journal of Biological Chemistry*, 287(50):42259–42268, 2012.

[3] J. Cursons, J. Gao, D. G. Hurley, P. R. Dunbar, M. D. Jacobs, E. J. Crampin, et al. Regulation of ERK-MAPK signaling in human epidermis. *BMC Systems Biology*, 9(1):41, 2015.

[4] P. M. Tan, K. S. Buchholz, J. H. Omens, A. D. McCulloch, and J. J. Saucerman. Predictive model identifies key network regulators of cardiomyocyte mechano-signaling. *PLoS Computational Biology*, 13 (11):e1005854, 2017.

[5] A. C. Zeigler, W. J. Richardson, J. W. Holmes, and J. J. Saucerman. A computational model of cardiac fibroblast signaling predicts context-dependent drivers of myofibroblast differentiation. *Journal of Molecular and Cellular Cardiology*, 94:72–81, 2016.
