# Supplementary material for: Cell signaling model for arterial mechanobiology
Source: PLoS Comput Biol. 2020 Aug 24;16(8):e1008161. doi: 10.1371/journal.pcbi.1008161 (PMC7470387; doi:10.1371/journal.pcbi.1008161)
Supplement: Attachment — Submitted filename: ArterialSignaling_ResponseToReviewers.pdf [file pcbi.1008161.s010.pdf]

We thank the reviewers for their assessment of our manuscript and helpful questions, comments, and concerns. Responses to individual comments and questions are provided below, and modifications are highlighted in red in the revised manuscript, which we feel clarified the presentation significantly.

Response to Reviewer 1

The authors present an excellent study in which they create a logic-driven computational model of the cell signaling network for growth and remodeling (G&R) of arteries. The authors based their network topology on a comprehensive literature review. Adjusting their parameters, they are able to validate their model by comparing predictions against different reports in the literature. The advantage of using a logic-driven approach is that the authors can validate their model based on qualitative behavior (e.g. a given output increases, stays constant, or decreases), which allows them to compare their predictions with a wide set of experiments from the literature. The authors then explore the main features of their network and perform a well thought out sensitivity analysis. Overall the article is excellent.

Thank you.

Major comments:
1. The authors present their cell signaling network, but are not specific about the cell population they model. At two points in the manuscript it seems that the regulatory network is specific for Smooth Muscle Cells (SMC), but at some other instances it is clear that they refer to endothelial cells. Most of the literature cited is indeed related to SMC response to mechanical input, but part of the network corresponds to endothelial cells, particularly nitric oxide (NO) dynamics and wall shear stress (WSS) mechanosensing. The distinction is important, since regulatory networks have cell-type specific components, but the network shown in Figure 1 mixes extra-celullar and intra-cellular signals and for the two different cell types. The article needs a clearer demarcation of the network components.

Thank you for this important comment. We have clarified and discussed this point in more detail. A long history of modeling the mechanics of the arterial wall has revealed that, though a multi-layered structure with different compositions defining the media and adventitia, considerable insight can be gained via “radial homogenizations” whereby the mean wall mechanics are captured well using a single-layered model. Similarly, we and others have shown that “growth and remodeling” simulations can capture well salient features of arterial adaptations and maladaptations using a radially homogenized model whereby the intramural cells are considered together. It is in the spirit of these prior mechanical models that we built this cell signaling model. That is, just as prior growth and remodeling simulations are based on shear stress mediated (endothelial mechanosensing) and intramural stress mediated (homogenized smooth muscle cell and fibroblast mechanosensing), so too the current model of signaling delineates only basic endothelial responses to flow and intramural cell (smooth muscle and fibroblast) responses to pressure-induced and AngII-induced stimuli. This point is now presented much more explicitly, in methods and discussion, the latter including interpretation, limitations, and future needs.

Response to Reviewer 2

This work presents a novel cell signaling network model to predict arterial smooth muscle cell responses to stimulation by mechanical stresses and angiotensin. Employing a logic-based, ordinary differential equation modeling approach that was previously developed for cardiac cell signaling, this current model predicts changes in signaling pathway activities and cellular outputs including matrix content, cell proliferation, and cell contractility. Excitingly, the model is able to correctly predict (qualitatively at least) a wide variety of input-output combinations reported in previous literature experiments, while also agreeing with several past studies of combined angiotensin + mechanical stimulation at the cell and tissue levels. The paper is very well written with a clear and rational organization, the findings are well supported and interesting, and the modeling approach (while previously used for cardiac cells) is innovative within the arterial mechanobiology field.

Thank you.

Minor comments:
1. Further clarification of smooth muscle cell vs. endothelial cell signaling:
The authors state in lines 45-47 that "… we designed our model to consider simultaneously the potential roles of altered wall stresses, wall shear stresses, and AngII infusion on changes in SMC phenotype…", and the authors also recognize in lines 61-62 that "wall shear stress is sensed primarily by endothelial cells…" Given these comments (and 'Remark 1' in the supplementary material), I presume that WSS-related reactions in the network are meant to capture endothelial cell production of NO & ET1 which then stimulate the rest of the network meant to capture smooth muscle cell signaling.

Thank you for this important comment, which is very similar to that by Reviewer 1. Hence, please see our response to Reviewer 1, noting that we have now clarified in many places that the model does indeed consider two primary cell responses, EC response to WSS leading to changes in NO and ET-1 and more detailed responses of intramural cells (SMCs and FBs) leading to many different associated cell signaling pathways. We have explained this in the text and in an improved presentation of the network diagram (Fig 1).

It is unclear, however, how this endothelial-smooth muscle cell interaction affected the model fitting and validation - in other words, were all WSS simulations compared to literature data from experiments that contained both SMCs & ECs (in vivo, ex vivo, in vitro co-culture)? Additional comments regarding these SMC-EC interactions in the main text (and perhaps Figure 1) could improve clarity on this point.

Regarding the WSS simulations - thank you, this is an important point to note in future refinements. Two of the relations were from thoracic aorta ex vivo, whereas the remaining were based on cell culture of a single cell type. The response of ET1 and NO to Wss was based on EC culture data, but we found the most common/only available cell culture data for the other relations to be from SMCs alone. We have noted in the limitations the need for a wider collection of co-culture data, which will also be important in model extensions if we are to consider layer-specific cell types and the associated crosstalk.

2. Criteria for network reaction rules:
A large number of past literature studies were used to assemble the network model structure, but it is unclear whether any particular criteria were used as thresholds to decide what rules to include/exclude. For example, were all of these studies performed with arterial smooth muscle cells, or were cells of any kind acceptable, etc.?

We have added more detail in the main text to explain the selection process. Our search focussed on vascular SMCs/FBs and 6 key pathways; clearly other pathways and interactions exist, but these six appear to be the primary pathways of importance for our focus on pressure, flow, and AngII stimulation. Reaction rules were included if there was direct evidence in studies of intramural vascular SMCs/FBs or aortic tissue samples (60 of the 82 rules). This formed the majority of the network, and where possible we used murine studies. In some cases, human or rat studies were used if comparable data were lacking for the mouse. To fully connect the network, some well-established reactions (such as the binding of a protein to its receptor, or widely known processes e.g. Ca=>MLCK, RhoA=>ROCK,…) were included, and we cited relevant review papers that discuss these more general phenomena (14 of 82 rules). Alternative cell types were considered if there was evidence across multiple cell types, and if these reactions had previously been proposed in vascular review papers (5 of 82 rules). The reactions for the wall shear stress are based on either murine tissue samples or aortic endothelial cells (2 of 82 rules). Finally, one rule (AngIIin=>AngII) was a model-specified reaction (1 of 82 rules). From this starting point, we envisage having to iteratively refine the network structure together with future experimental studies. Importantly, the rule-based approach provides a convenient means to do this. These are points that we have made sure to emphasize.

3. WSS and AngII baseline assumptions:
Equation 2 lists yWss baseline equal to 0.5, and Equation 3 lists yAngIIin baseline equal to zero. It seems unclear why these two baseline values are not set as the parameter b, which is used as the baseline parameter for yStress and other cell receptors. Clarifying the justification and implication for these assumptions could help support this choice.

The input species `AngIIin' represents exogenous sources of AngII, which are only present in certain experimental protocols, as, for example, chronic infusion of AngII via a mini-osmotic pump. We thus set it to

zero in our baseline case because this represents our control state of the artery, to which perturbations are applied (exogenous AngII being one such perturbation). Note that this does not mean that there is no AngII signaling at baseline, since AngII is also activated by intramural stress.

The baseline for wall shear stress (Wss) was a modeling choice due the desire to study both increased and decreased Wss equally, and based on examination of the corresponding ET1 and NO steady states (so that ET1 increases below baseline Wss, and NO increases above target Wss). We have included additional clarification and discussion of these model assumptions in the text.

4. Knockdown simulations:
In Figure 5, a few of the knockdown simulations (e.g., Arp23, Cdc42, FAK) produced no changes in any of the nodes (including the knocked-down nodes themselves). This seems surprising and worthy of further explanation (particularly for FAK, which is connected to a number of downstream nodes).
We thank the reviewer for this astute observation, which requires further comment. The reason for the apparent lack of changes are that the species mentioned already had low basal values, and their absolute values were modulated only slightly when enforcing Y_{max}=0.1. These differences in absolute values are present, but were not visible on this scale. We now explain this in the text and include a supplementary figure that shows this subset of results (FAK, Cdc42, Arp2/3, ActomyosinActivity) separately, using a colorscale with a smaller range.

Response to Reviewer 3

The authors use a network to analyze how arteries grow and remodel in response to factors like wall stress, angiotensin II and pressure. This is to study both healthy and maladaptive responses in vessels using signaling pathways. They have found good qualitative agreement of the model with papers previously published about the system and did a parameter sweep to optimize values for parameters like n and EC50.

However, the manuscript does not convey clearly how the model can be used to study the system being described. It is not clear what cells signaling mechanisms of the arterial wall are being considered in this study. As noted below, the figures do not have clear conclusions one can draw from them, nor is it clear how model validation was conducted. In addition, the paper makes biological conclusions without acknowledging limitations of the model, and does not adequately describe how this cell signaling network can represent a vessel undergoing growth and remodeling.

We appreciate these comments, which revealed the need to clarify significantly many aspects of our presentation, both text and figures. Please see below our response to each question/concern.

Major comments:
1. It is ambiguous what system this cell signaling model applies to. Is the model supposed to be representing all of the vessel wall? Do SMCs have a role in ECM deposition? Is this a model taking into account multiple cell types? It is hard to see how Figure 1 can be applied to the entire vessel wall when there are cell receptors in the model.

Thank you for this important question, which is very similar to that raised by Reviewer 1. As noted in our response to her/him, please note that: A long history of modeling the mechanics of the arterial wall has revealed that, though a multi-layered structure with different compositions defining the media and adventitia, considerable insight can be gained via “radial homogenizations” whereby the mean wall mechanics are captured well using a single-layered model (Humphrey, 2002 - book). Similarly, we and others have shown that “growth and remodeling” simulations can capture well salient features of arterial adaptations and maladaptations using a radially homogenized model whereby the intramural cells (SMCs and FBs, noting that in elastic arteries, such as we are interested in, the SMCs exhibit a “matrix phenotype” as described by R. Mecham et al. whereby SMCs are primarily responsible for producing, maintaining, and remodeling the matrix) are considered together. It is in the spirit of these prior mechanical and mechanobiological models that we built this cell signaling model. That is, just as prior growth and remodeling simulations are based on shear stress mediated (endothelial mechanosensing) and intramural stress mediated (homogenized smooth muscle cell and fibroblast mechanosensing) changes, so too the current model of signaling delineates only basic endothelial responses to flow and intramural cell (smooth muscle and fibroblast) responses to pressure-induced and AngII-induced stimuli, which mainly affect matrix. This point is now presented much more

explicitly, in methods and discussion, the latter including interpretation, limitations, and future needs. Thank you.

2. To better illustrate the model prediction capabilities, Figure 2 would have to include the direct comparison to model results. Addressing this will help emphasize the capabilities of the model, which remain unclear.

We agree that the original presentation forced the reader to compare the results of Figure 2 against the tabulated results from the literature, which was cumbersome. We now have combined these two “figures” to allow direct comparison, and provide in each of the blocks in Figure 2 a “check mark” to denote model agreement and a “x” to denote model disagreement, with an explicit note to refer the reader to the table of experimental results. This should improve presentation considerably, thank you.

3. Model validation details are missing in the manuscript. It is unclear whether the set of 37 papers were used for construction or only for validation. This is an important component of the model that should be included in the methods

Agreed, we have now clarified this. Independent sets of papers were used for construction and validation, with network construction focused on specific reactions (i.e. edges) and validation based on input-output relations (i.e. propagation through the network). This is an important point, now clarified.

4. It is unclear what the importance of Figure 3 is. The steady state behavior described in the figure does not add much on their own and the 3D graphs are confusing to interpret. Unless the authors find the need to further describe the contribution of this state to the G&R modeling efforts, it is not clear that this figure should be included.

We have moved this figure to the Supplementary Material, as the key points can be understood from the figure that followed it. Showing absolute, rather than fold-change, steady state behaviors does help in understanding the reasons behind the transition from increasing (>1) to decreasing (<1) fold-changes, where we wanted to use this figure to highlight the role of non-monotonic surfaces. However, this point can equally be made in the Ruddy case study, and we have shown (a reduced number of) 3D surfaces here instead. By using a specific example, we are able to better explain their purpose (as a dose-response surface, for two different input variables), and we made sure to better demonstrate their interpretation using cross-sections with an analysis of a specific species (MMP9). Thank you for the suggested clarification.

5. Figures 6 and 8 could be annotated to show how these model prediction results compare to qualitative experimental data.

Fig 6 (original labeling) is presented in a way that can be directly compared to experimental results by Ruddy *et al.* (2009), with arrows corresponding to trends they observed. We have made this clearer in the figure caption, by describing this feature and directing the interested reader to the relevant figures in the original paper, which we do not include in the manuscript because they are from previously published material. We have also significantly edited our presentation of this case study in the text (also including an additional figure), however, to better describe the qualitative similarities and differences we obtain, and the resulting conclusions that can be drawn from this initial study.

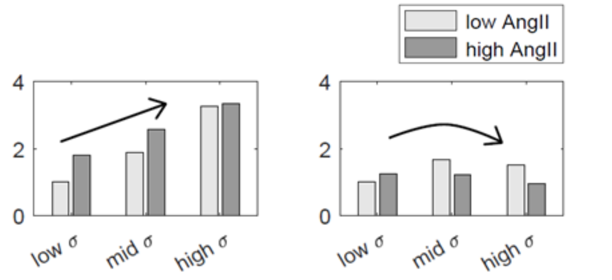

Model MMP predictions with optimized default parameters

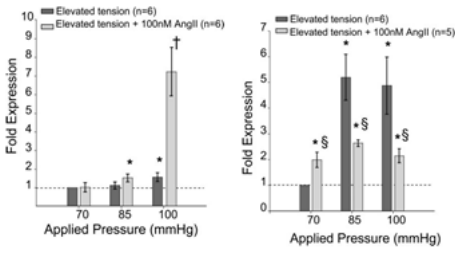

Ruddy et al, *Circulation,* 2009

Fig 8 (original labeling) aims to simulate the study by Wu *et al.* (2014). The most effective presentation of this case study is a direct comparison, as included below. We do not do this in our manuscript, but instead we have added experimental datapoints to Fig 8 where available, although the numerical values with the p38 MAPK inhibitor were not provided in their paper. These values are shown in bar graphs in Fig 5D,E in the cited paper (also included here), which we have emphasized in our figure caption for the interested reader. Note that our presentation in the manuscript differs to that of the original paper (and the figure included here) since, when not comparing directly, we felt this was a more concise summary of the results.

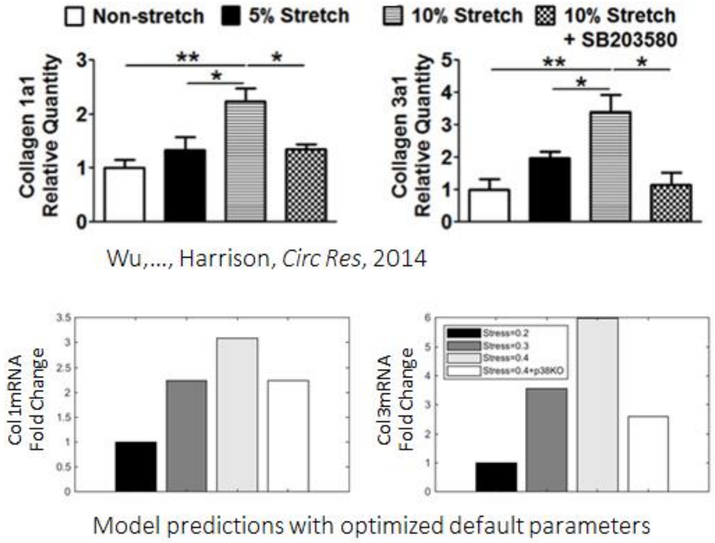

6. In the Discussion Section, it is not clear what biological conclusions can be made based on this model since the Saucerman model is normalized from 0 to 1. Is there enough data in the literature to make strong conclusions about what the model agrees with and does not? There are contradictory experiments, what does that mean for the confidence in the model?

Different (types of) models have different utilities, different meanings. The advantage of this logic-based approach is that one can predict well myriad trends and "fold-changes", which often is all that is available from the many different biological assays available. An advantage of the 0-1 range is that one can capture the general trends but also the quantitative outputs (when available) via appropriate scaling. In this paper, given the lack of detailed quantitative data in the literature, we did not perform a scaling. In addition, we emphasize that one advantage of (all) computational models is that they can be used to design appropriate experiments, effectively revealing what should be measured and why. With regard to the contradictory experiments, this does make model validation challenging, but we must start with the data available. Whilst there are many possible reasons for these conflicts, one possible explanation is in fact provided by our model, which is that the response of some species to perturbations can be non-monotonic, meaning that the qualitative outcome is sensitive to baseline conditions and perturbation magnitudes (illustrative explanation in Fig S4). We think that this highlights a point of key importance to these types of fold-change experiments; often only one measurement is made, but for complex, nonlinear responses, we can only characterize the behavior when multiple samples are taken.

We have discussed the limitation of normalizing species to [0,1], with a focus towards future quantitative studies when data is available. We have expanded on the biological importance of our qualitative results, with a focus towards experimental design (suggesting the use of multiple doses and controlled baselines, which would enable a better understanding of whether conflicting fold-change results are a result of non-monotonic responses). Thank you for this key comment.

7. In general, there is not much discussion of the limitations of this type of modeling to study this system It is not clear how the species were chosen, what is the scope of the G&R model, and what can this model be applied to. Addressing these questions will significantly improve the manuscript.

Agreed, we have now addressed limitations better as well as providing some insight into future needs.

8. To add model significance, the work could benefit from more inhibition studies with time courses to compare against.

Agreed, though evaluating nearly 100 papers, the difficulty is finding appropriate time course data in the literature, which is surprisingly scant along the lines needed here. Again, this need for data (limitation of the literature) is now noted.

9. The authors propose to use this cell signaling model for growth and remodeling. However, it is not clear where the feedback in the signaling model comes into play. How are the authors accounting for the constant adapting and remodeling of the vessels?

Thank you for this astute observation. We have now added a discussion (limitation and future work) to detail what we envision, namely to couple signaling models such as this with "G&R" models to create mechanistic multiscale models.

Minor comments:
1. Figure 1 could be made more clear by differentiating cell signaling molecules from receptors vs transcription factors vs mRNA, etc.

Thank you for this important suggestion. We have improved the clarity of Fig 1 by distinguishing extracellular, transmembrane and intracellular components, and by showing the EC and SMC/FB components explicitly.

2. MMP9 is also involved in ECM remodeling, thus is there a reason MMP9 does not have the same effect as the other MMPs?

Here we consider MMP1, MMP2 and MMP9, where MMP1 is a collagenase and MMP2 and MMP9 are gelatinases, although these can also affect collagen and ECM remodeling as noted by the reviewer. The action of the different MMPs on collagen types I and III (considered here) were based on several sources, one of which being the well-regarded review of Nagase et al. They write, "MMP2 and MMP9 digest a number of ECM molecules including type IV, V and XI collagens, laminin, aggrecan core protein, etc. MMP2, but not MMP9, digests collagens I, II and III in a similar manner to the collagenases." From this point, evidence was found of the action of MMP2 on collagens I and III, but no evidence was found for MMP9 acting on these specific types of collagen. Perhaps this is an action that requires further investigation in future refinements of the model, where we will also consider additional collagen subtypes. Note that MMP9 does act similarly to MMP2 in the activation of latentTGFb, and in this model this is its primary downstream role. We have added in the discussion that many other species of matrix proteins/glycoproteins/GAGs and MMPs/ADAMPTS, etc. can and should be added in future models, with more detailed consideration of their interactions becoming necessary. Of course, the more complex a model, the more difficult it becomes to validate it. We felt that the current level of detail, given the key papers found when mining the literature, represented a good compromise between complexity and assessment.

3. Figure 5 is missing labels: the color bar is not labelled, the y-axis is not labelled, and "knockdowns" is perhaps not a great descriptor of the x-axis since these are specific nodes that are knocked down.

Corrected, thanks.

4. The Figure 7 color bar is missing its label.

Corrected, thanks.

5. Section 2.3 could better be described as a sensitivity analysis rather than "knockdowns"

We have made the suggested change.

6. Was there an attempt to vary the parameter of tau? If not, it should be justified and described as a limitation.

We did not vary tau for these simulations, due to lack of appropriate knowledge about the time-dependent behaviors of each species. This is indeed an important limitation that needed highlighting, and we have now done so in the methods and discussion. Thanks.
