# Supplementary material for: Cell signaling model for arterial mechanobiology
Source: PLoS Comput Biol. 2020 Aug 24;16(8):e1008161. doi: 10.1371/journal.pcbi.1008161 (PMC7470387; doi:10.1371/journal.pcbi.1008161)
Supplement: Attachment — Submitted filename: ArterialSignaling_ResponsetoReviewers2.pdf [file pcbi.1008161.s011.pdf]

We thank the reviewers for their further assessment of our manuscript, which led to additional clarifications. Responses to individual comments and questions are provided below, and modifications are highlighted in red in the revised manuscript.

Response to Reviewer 1

I would like to commend the authors for this nice work. My main concern in the initial version of the paper was the lack of specification about the different cell types in the regulatory network model. In my experience, regulatory networks are cell-specific and I raised that concern before. However, the authors argue that models of growth and remodeling (G&R) of the artery homogenize the different layers into a single set of phenomenological equations. Therefore, a homogenized regulatory network would indeed be a natural way to couple to the author's G&R framework to this, more mechanistic model. While I think there is still room for further development with more detail on the regulatory network, dividing into the different cell types, I understand the authors' position and recognize that the revised version has several clarifications about this homogenization and its limitations. Therefore I am satisfied with the revised version of this manuscript.

Thank you.

Response to Reviewer 2

This revised manuscript presents a novel cell signaling network model to predict arterial smooth muscle cell responses to stimulation by mechanical stresses and angiotensin. The revision is highly responsive to the reviewer comments, addresses all my concerns, and has further improved into an even more excellent study.

Thank you.

Response to Reviewer 3

The authors' revisions have generally strengthened the manuscript and show better comparison of their model to existing data. Their reworking of Figure 1 and explanation shows a clearer picture of the system they are trying to model. The additions to the discussion help acknowledge the limitations of this type of model.

Thank you.

Major comments:

1. While the authors have responded to our comments, this reviewer finds that the methods of the model construction and qualitative validation carried out lack important details that need to be addressed.
It would be more clear if authors can indicate what the inclusion of 37 independent papers were used for. As of right now, it is not clear if these papers were used for parameterizing the model after using the 72 studies to construct the model or purely for validation.

Thank you for this additional question; its clarification will be important to future readers. The 37 independent papers were used for model parameterization. Model parameters were tuned such that the model input—output relations best agreed with corresponding relations from those experimental studies (shown in S2 Appendix), thus validating the utility of the model. We have now described this in more detail in the main text and caption related to Fig 2, and in the methods.

2. The authors describe the parameters as uniform across the network. It would be best if they specified whether the parameters were set to the same initial values and then perturbed independently for each species. Otherwise, it is unclear if the authors assume a uniform distribution of the parameters rather than a single default value.

There are single default values for the parameters described as 'uniform', and these values are used for all reactions. We have now clarified this in the text. This approach is consistent with that used by Saucerman's group in multiple papers, with similar goodness here.

3. Given that the authors do not include the system of differential equations and thus do not define tau, they should refer to the original paper where the equations are presented for the reader to reproduce their results.

Good point. We now refer again to the original paper but also point more directly to S3 Appendix, in which we presented an illustrative example, including construction of logic statements, derivation of the corresponding governing equations (including tau) and simple analyses.

4. It would be useful to describe in more detail how the parameter p is perturbed - did the perturbation follow a distribution (i.e, uniform distribution between 0 and 1, Gaussian, etc)?

p is a constant value to be added to the baseline input value, which we have now clarified in the text.

5. It is unclear what the criteria are for the use of "best data available" in page 3 line 45. It would be important to include this in the text.

Thank you, we now describe this as the most complete data available (to our knowledge, following extensive literature search), which consider multiple perturbed inputs and multiple perturbation magnitudes.

6. While the reviewers appreciate the authors trying to connect this signaling network model to their tissue-level mechanics, and thus focus on the global, mean, arterial mechanics (homogeneous walls), it is important to clarify that the fibroblasts are primarily adventitial fibroblasts rather than circulating fibroblasts such as it is done in the case studies.

Thank you for this important comment as well. Again, we "homogenize" the intramural cellular response consistent with the radial homogenization that is used in most continuum models of the vascular wall as well as much of the biological data available from "bulk" PCR, Westerns, and RNAseq (all based on wall homogenates, particularly for murine vessels). As such, the intramural cells in our model can collectively exhibit a "contractile phenotype", a "proliferative phenotype", and/or a "matrix phenotype." Most data to date have focused on the importance of the matrix phenotype in hypertensive remodeling, meaning the synthesis/deposition and degradation/removal of matrix. We loosely stated in the paper that smooth muscle cells and fibroblasts are the two primary cell types exhibiting this "matrix phenotype." The reviewer is correct in pointing out, however, that there is need for more clarity. Whereas modest (adaptive) changes in wall thickness can likely be achieved by resident cells – phenotypically modulated smooth muscle cells, fibroblasts, and possibly activated resident progenitor cells (the Sca1+ cells in the outer media) – in cases of aortic fibrosis (largely adventitial), additional bone marrow-derived cells can be recruited and contribute to the matrix accumulation. These "fibrocytes" are probably the "circulating fibroblasts" to which the reviewer refers. We had not thought it appropriate to go into this detail / complexity given our initial homogenization, but we agree that it should be noted, especially in limitations (needs for future extensions). This is now done, thank you. We refer primarily to a prior study in which we were involved that delineates this carefully – Harrison group at Vanderbilt. Thank you.

7. The authors revised figures and their caption and made them more clear in the revised version. However, Figure 3 is not well justified and could be moved to the supplement. Figure 8 is also not well justified to be included in the methods as its results are not unique to the paper.

We have removed Fig 8 and moved Fig 3 (with discussion/analysis) to an Appendix.

Minor comments:
1. Page 17, line 398 'lead' should read 'leads'

Corrected.

2. Page 19, line 442 - The authors refer to using a Hill approach but this could be rephrased as it is not completely clear what they mean.

Agreed, we have rephrased this sentence.

3. Figure 5: The addition to the caption is worded confusingly and could be improved with more clarity, is it referring to the first two dose-dependent qualitative behaviors or the baseline and first dose-dependent behavior? Also, best if the authors only included a few panels to emphasize important results and moved the rest to the supplement.

We have removed the confusing “baseline and dose-dependent” wording in the caption, since we can better describe this dependence in the main text. We have moved the TGFb1 and TSP1 panels to a supplementary file.

4. Figure 7: Since data from the citation 52 are included in the AngII model predictions, it would be more clear to include the data for -p38MAPK. These data can be found via tools such as WebPlotDigitizer.

Thank you, we have included the data for –p38MAPK.
