# Supplementary material for: Cell signaling model for arterial mechanobiology
Source: PLoS Comput Biol. 2020 Aug 24;16(8):e1008161. doi: 10.1371/journal.pcbi.1008161 (PMC7470387; doi:10.1371/journal.pcbi.1008161)
Supplement: Attachment — Submitted filename: ArterialSignaling_ResponsetoEditors.pdf [file pcbi.1008161.s012.pdf]

Response to editors

In order for this paper to reach its potential, it is important that the model is fully available for others to test and extend. The data availability statement currently states that "All relevant data are within the manuscript and its Supporting Information files". However, the actual data and code are not included in the Supplement. We would like to see these included either in the supplement, or preferably in a repository.

We have added a link to a GitHub repository (https://github.com/irons-l/arterialsignaling), which contains the necessary code for testing and reproducing model results from the main text. These codes are based on the open-source `Netflux' code available at: github.com/saucermanlab/netflux, as cited throughout the manuscript and in our repository README and scripts.
